# The Nubian Complex of Dhofar, Oman: An African Middle Stone Age Industry in Southern Arabia

**DOI:** 10.1371/journal.pone.0028239

**Published:** 2011-11-30

**Authors:** Jeffrey I. Rose, Vitaly I. Usik, Anthony E. Marks, Yamandu H. Hilbert, Christopher S. Galletti, Ash Parton, Jean Marie Geiling, Viktor Černý, Mike W. Morley, Richard G. Roberts

**Affiliations:** 1 Institute of Archaeology and Antiquity, University of Birmingham, Birmingham, United Kingdom; 2 Archaeological Museum, Institute of Archaeology, National Academy of Sciences of Ukraine, Kiev, Ukraine; 3 Department of Anthropology, Southern Methodist University, Dallas, Texas, United States of America; 4 School of Geographical Science and Urban Planning, Arizona State University, Tempe, Arizona, United States of America; 5 Department of Anthropology and Geography, Oxford Brookes University, Oxford, United Kingdom; 6 Institut für Naturwissenschaftliche Archäologie, University of Tübingen, Tübingen, Germany; 7 Institute of Archaeology of the Academy of Science, Prague, Czech Republic; 8 Centre for Archaeological Science, School of Earth and Environmental Sciences, University of Wollongong, Wollongong, Australia; University of Oxford, United Kingdom

## Abstract

Despite the numerous studies proposing early human population expansions from Africa into Arabia during the Late Pleistocene, no archaeological sites have yet been discovered in Arabia that resemble a specific African industry, which would indicate demographic exchange across the Red Sea. Here we report the discovery of a buried site and more than 100 new surface scatters in the Dhofar region of Oman belonging to a regionally-specific African lithic industry - the late Nubian Complex - known previously only from the northeast and Horn of Africa during Marine Isotope Stage 5, ∼128,000 to 74,000 years ago. Two optically stimulated luminescence age estimates from the open-air site of Aybut Al Auwal in Oman place the Arabian Nubian Complex at ∼106,000 years ago, providing archaeological evidence for the presence of a distinct northeast African Middle Stone Age technocomplex in southern Arabia sometime in the first half of Marine Isotope Stage 5.

## Introduction

### The Nubian Complex

**Figure 1:**
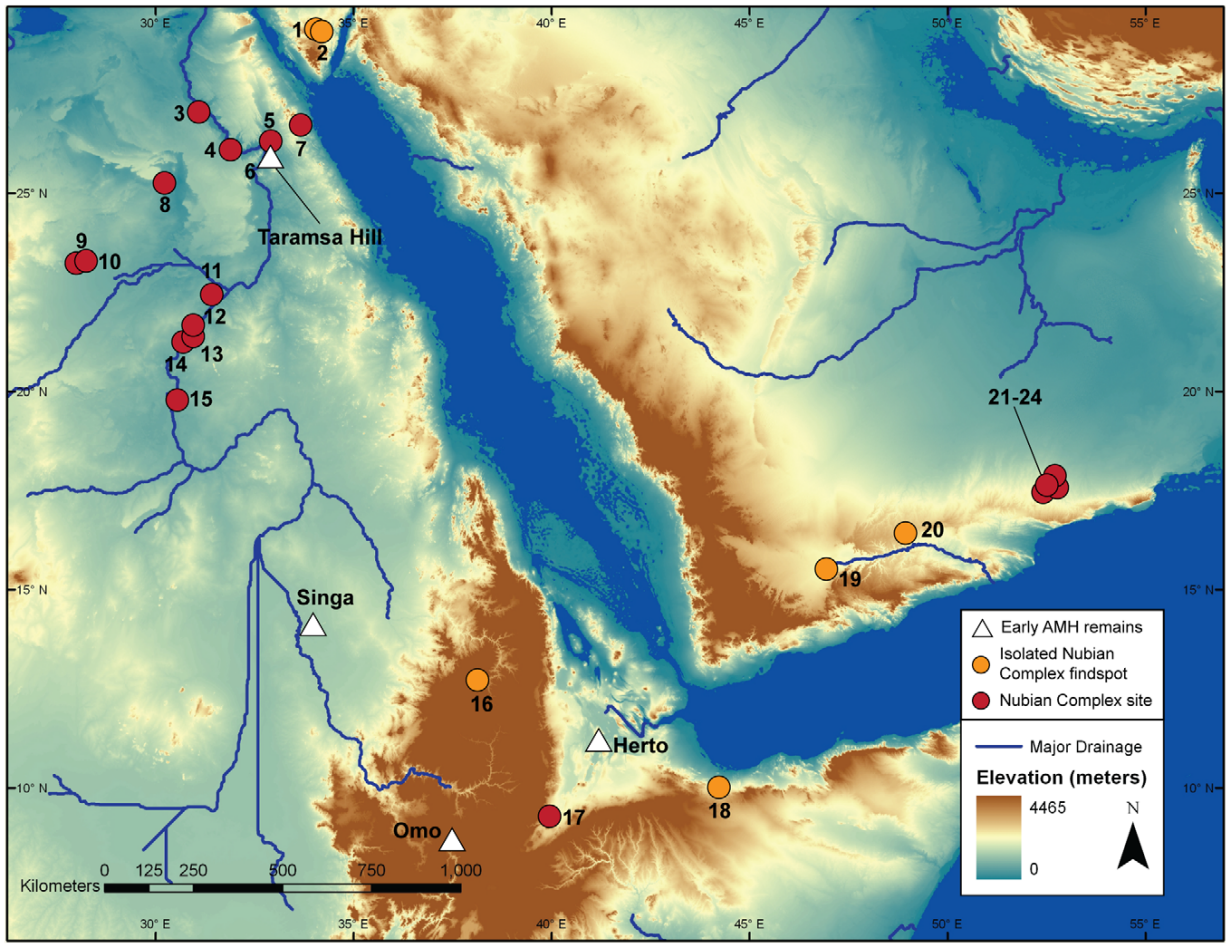
Map of Nubian Complex occurrences in Northeast Africa and Arabia. Distribution of Nubian Complex sites and findspots are depicted, as well as MSA/MP sites with human remains. To account for shoreline configuration ∼100 ka, sea level is adjusted to −40 m below present levels. Nubian Complex sites include: Jebel Urayf (1), Jebel Naquah (2), Nazlet Khater (3), Abydos (4), Makhadma (5), Taramsa Hill (6), Sodmein Cave (7), Kharga Oasis (8), Bir Tarfawi (9), Bir Sahara (10), Abu Simbel (11), Jebel Brinikol (12), 1035 (13), 1038 (14), Sai Island (15), Gorgora Rockshelter (16), K'One (17), Hargeisa (18), Shabwa (19), Wadi Wa'shah (20), Aybut Al Auwal (21), Aybut Ath Thani (22), Mudayy As Sodh (23), and Jebel Sanoora (24).

The Nubian Complex is a regionally distinct Middle Stone Age (MSA) technocomplex first reported from the northern Sudan in the late 1960 s [1], [2]. Archaeological sites belonging to the Nubian Complex (Fig. 1) have since been found throughout the middle and lower Nile Valley [3]–[6], desert oases of the eastern Sahara [7], [8], and the Red Sea hills [9], [10]. Numerical ages from Nubian Complex sites (Table 1) are constrained within Marine Isotope Stage 5 (MIS 5), although temporal differences have been observed among assemblages; as such, it is divided into two phases, an early and a late Nubian Complex [5], [11].

**Table 1:** Numerical ages of Nubian Complex sites in Africa and Arabia.

Site	Location	Age	Method	Reference
Aybut Al Auwal	Nejd plateau, Oman	106±9	OSL	
Sodmein Cave	Red Sea hills, Egypt	119±18	TL	[10]
Taramsa Hill	Lower Nile Valley, Egypt	74±4; 103±8	OSL	[21]
Sai Island	Middle Nile Valley, Sudan	<162	OSL	[4]
Bir Tarfawi/Bir Sahara - Gray Lake Phases 1 & 2	Eastern Sahara, Egypt	∼105±23	OSL, TL, ESR, U-series, AAR	[7]
Bir Tarfawi/Bir Sahara - Green Lake Phase	Eastern Sahara, Egypt	∼114±10	OSL, TL, ESR, U-series, AAR	[7]
Mata'na Site G, Kharga Oasis	Eastern Sahara, Egypt	>103±14	U-series	[8]
Bulaq Wadi 3, Kharga Oasis	Eastern Sahara, Egypt	>114±4	U-series	[8]

Dating method abbreviations are: radiocarbon (_14_C), thermoluminescence (TL), optically stimulated luminescence (OSL), electron spin resonance (ESR), _230_TH/_234_U (U-series), and amino acid racemization (AAR).

**Figure 2:**
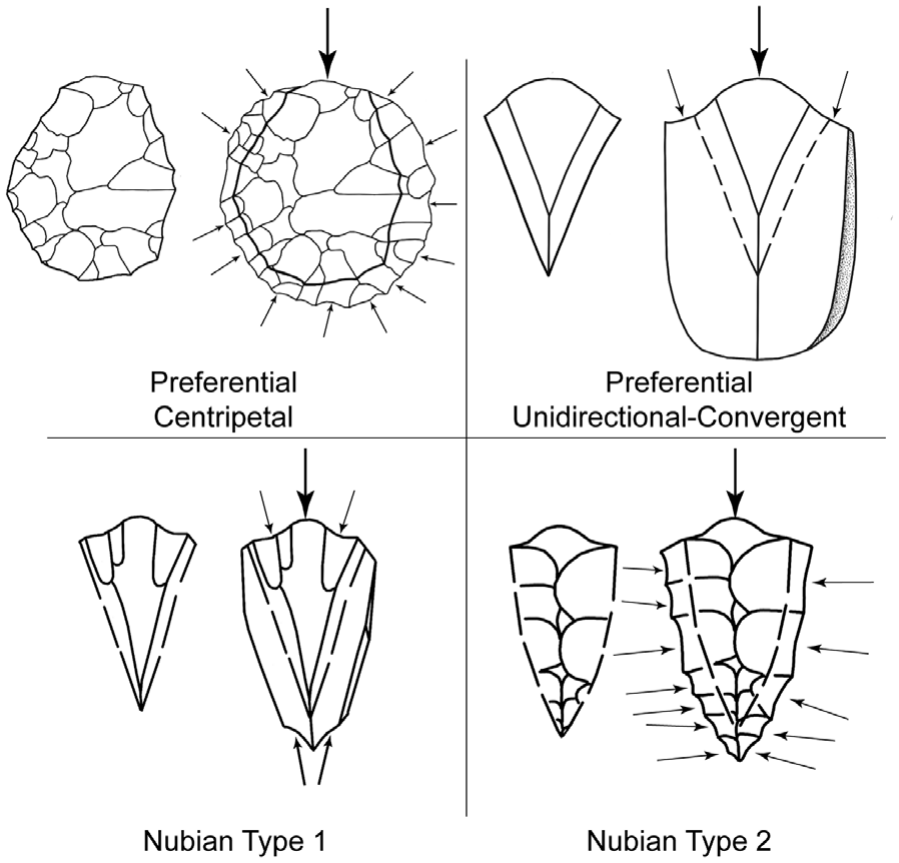
Schematic of preferential Levallois core preparation strategies mentioned in text.

Nubian Complex industries are distinguished by a characteristic and highly standardized method of preferential Levallois reduction, “mass-produced from an elaborate archetype” [1]. Nubian core technology is considered a regional variant of the preferential Levallois method for producing points, sensu [12], recognized by its triangular/sub-triangular shaped cores and a specific opposed platform preparation of the primary working surface, from which Levallois blanks are struck [13]. There are two sub-types of Nubian Levallois core preparation, referred to as Nubian Type 1 and Type 2 (Fig. 2). The primary working surface of a Nubian Type 1 core is formed by two distal-divergent removals creating a steeply angled median distal ridge, in order to set up the core for the preferential removal of an elongated and pointed flake or blade. Although the end product is the same, the steep median distal ridge on a Nubian Type 2 core is achieved through bilateral shaping of the primary working surface. These two methods are not mutually exclusive; in some instances, the primary working surface of the Nubian core exhibits a combination of partial-distal and lateral shaping. In every case, Nubian cores have highly characteristic preparation at the distal end of the core to create a steeply peaked triangular cross-section, which results in the signature Nubian Levallois point [1], [13]. Nubian Levallois core preparation strategy is technologically dissimilar to the Levallois point-producing industries found at nearby Levantine Middle Palaeolithic (MP) sites, which are broadly characterized by preferential unidirectional-convergent and centripetal reduction systems [14]–[19].

The early Nubian Complex is distinguished by a higher frequency of Nubian Type 2 cores in conjunction with bifacial foliates and handaxes [4], [20]. The late Nubian Complex, on the other hand, shows a predominance of Nubian Type 1 cores and a complete absence of bifacial reduction [5]. Late Nubian Complex assemblages have been found in stratigraphic succession overlying early Nubian Complex horizons at Sodmein Cave [11] and Taramsa Hill 1 [21] in Egypt; in both cases separated by a chronological hiatus. The early Nubian Complex roughly corresponds to early MIS 5, while numerical ages for the late Nubian Complex in northeast Africa fall in the latter half of MIS 5.

Taking into account its distinct, regionally-specific characteristics, Marks [2] notes that the Nubian Complex has no exogenous source and, therefore, probably derives from a local Nilotic tradition rooted in the late Middle Pleistocene (∼200–128 ka). This supposition is supported by the early Nubian Complex assemblage at Sai Island, northern Sudan, which overlies a Lupemban occupation layer dated to between ∼180 and 150 ka. The archaeological sequence shows an increase in the use of Nubian Levallois technology over time, concurrent with a reduction in both the size and frequency of Lupemban bifacial foliate tools. From this seemingly continuous technological continuum, Van Peer and Vermeersch [5] conclude, “the Nubian Complex represents a changed Lupemban lithic technology.” As it appears to derive from the Nilotic Lupemban industry of Levallois facies [20] - the northernmost extension of a sub-Saharan industry - the Nubian Complex is now classified as Middle Stone Age (African), rather than Middle Palaeolithic (European and Near Eastern).

There are claims for the presence of Nubian technology in eastern Arabia. One “possible” Nubian Type 1 core was reported at Jebel Barakah, UAE, illustrated in Wahida et al. [22]. Since it is neither triangular/sub-triangular, nor does it exhibit the essential steep triangular distal guiding ridge, we reject the validity of this attribution and note it is the only specimen described as Nubian within an otherwise entirely preferential centripetal Levallois reduction strategy.

Despite published reports of Nubian [23] or “Nubian-like” [24] technology in the Levant, the purported presence of Nubian Levallois reduction at Rosh Ein Mor, Tor Faraj, Tirat-Carmel, Yabrud, Skhul, Qafzeh, and Biqat Quneitra is largely unsubstantiated. Occasional cores with bidirectional preparation do not signify the presence of Nubian Complex technology, following the formal definitions of Guichard and Guichard [1] and Van Peer [13]. While there is overlap between Nubian Type 2 core preparation and some preferential point-producing Levallois reduction systems in the Levantine Mousterian, eg. [25], the Nubian Type 1 technological variant is not present north of the Sinai. In their analysis of the late Levantine Mousterian assemblage from Kebara, Meignen and Bar-Yosef [26] arrive at a similar conclusion: “For instance, at Kebara, triangular blocks are the common morphology encountered. This morphology is determined by the way the removals are organized on the core. But other dispositions are possible, in particular through opposite and diverging removals, known as the ‘Nubian’ method. This pattern never occurs at Kebara.”

At present, the northernmost extent of Nubian Type 1 cores is demarcated by assemblages found in the vicinity of Jebel Urayf and Naquah, in central-east Sinai [27]. As for its southern distribution, Nubian Levallois technology has been reported in the Horn of Africa. Excavations at K'One Crater [28] and Gorgora Rockshelter [29] in Ethiopia produced assemblages with Nubian Levallois cores. Of particular note, given the proximity to the Bab al Mandeb Strait, two cores from an alluvial section near Hargeisa, northern Somalia are illustrated in Clark [30], which exhibit Nubian Type 1 Levallois preparation. The first hint of the Nubian Complex extending into southern Arabia was documented by Inizan and Ortlieb [31], who illustrate three cores from Wadi Muqqah in western Hadramaut, Yemen, with Nubian Type 1 and Type 2 technological features. More recently, Crassard [32] presents a handful of Levallois point cores exhibiting Nubian Type 1 preparation from Wadi Wa'shah, central Hadramaut, Yemen.

In light of these tantalizing, yet inconclusive findings, the Dhofar Archaeological Project (DAP) was initiated in 2010 to explore the Late Pleistocene archaeological record of the Dhofar region in southwestern Oman. During the 2010 fieldwork campaign, a surface scatter with Nubian Type 1 and Type 2 Levallois cores was discovered in Wadi Aybut, central Dhofar. Subsequent research by DAP has focused on geoarchaeological investigation of the Aybut drainage system and surrounding landscapes, optically stimulated luminescence (OSL) dating of cemented fluvial sediments at Aybut Al Auwal that contained a handful of diagnostic Nubian Levallois artifacts, systematic survey to articulate the distribution of Nubian Levallois core technology throughout Dhofar, and techno-typological analysis of Nubian Levallois reduction strategies in Dhofar to assess the relationship of these assemblages with the African Nubian Complex.

### Environmental Context

**Figure 3:**
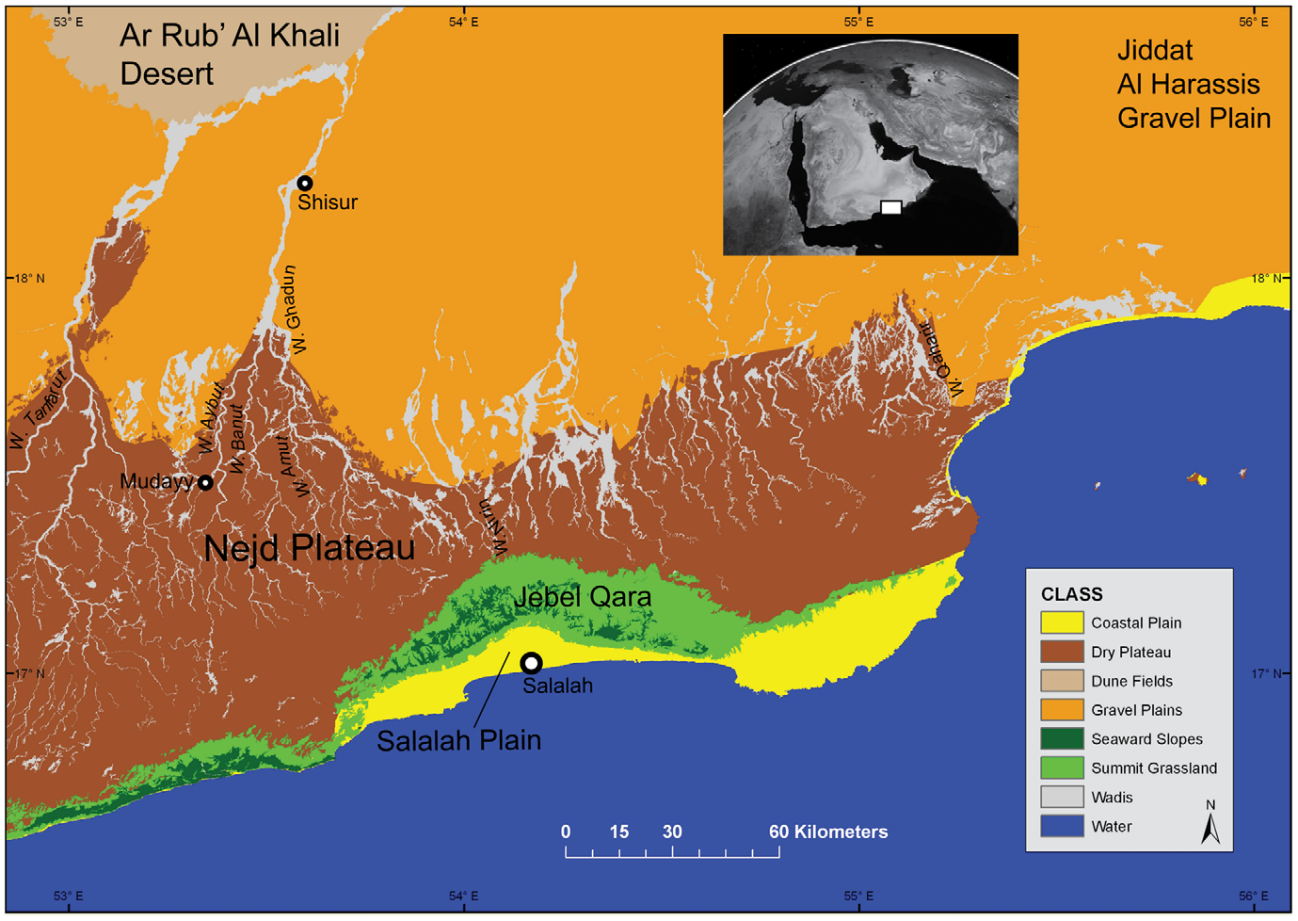
Dhofar ecological zones and place names mentioned in text.

Dhofar is situated in the southwestern corner of Oman, covering an area of nearly 100,000 km^2^. The landscape of this region encompasses a variety of geomorphic settings, partitioned into four ecological zones (Fig. 3): 1) Salalah coastal plain, 2) Jebel Qara escarpment, 3) Nejd plateau, and 4) Rub' Al Khali desert [33].

The coastal plain, stretching some 90 km in length, is a crescent-shaped landmass up to 15 km wide, bounded by the Arabian Sea and the Jebel Qara escarpment. The plain is composed of alluvial fans up to 100 m thick, which are cut by short wadis draining southward from the escarpment toward the sea. Several springs emerge along the foot of the escarpment, around which grow date and coconut palms, bananas and other tropical fruits, and grasses [34].

North of the plain, the Jebel Qara-Jebel Samhan mountain chain rises abruptly to a maximum height of 850 m above sea level. The high escarpment forms an orographic barrier that traps moisture from the Indian Ocean Monsoon (IOM), generating up to 200 mm of annual rainfall across the mountains. Due to moisture brought by the IOM, the Dhofar highlands are covered in a mantle of dark brown clay soil that supports a subtropical cloud forest belonging to the Somalia-Masai center of endemism [35].

Northwards, past the current watershed divide, the escarpment flattens out onto a deeply incised limestone plateau called the Nejd, which extends approximately 250 km east-west and 150 km north-south. Around its southern extent, the Nejd is a barren scabland marked by an intricate series of minor wadis dissecting the plateau. These smaller drainage systems converge into larger and more deeply incised canyons that extend up to 100 km northward across the plateau, running parallel to one another. As they reach the northern extent of the Nejd, the wadis empty onto a gently undulating gravel plain flanking the Rub' Al Khali desert.

Three high-quality Eocene chert beds outcrop widely throughout the Nejd plateau, making the landscape appealing for prehistoric toolmakers. Fine-grained, large, banded chert slabs are found within the *Mudayy* member, which is the highest quality on the plateau, outcropping in the southern and central regions. Chert-bearing units within the overlying *Rus* formation are concentrated in the southern Nejd, including the lower chalky *Aybut* member and upper marly-carbonate *Gahit* member. The quality and size of this raw material is quite variable; due to post-depositional displacement, most of the *Aybut* member chert is highly fractured. Thin, high-quality grey chert plaquettes are found within the *Gahit* member [34]. Regardless of size or dimensions, evidence indicates that Nubian Complex toolmakers in Dhofar were able to construct Levallois cores from all three chert types. The quality of raw material in terms of flaking properties, degree of internal fracturing, and mineral inclusions, however, does seem to have significantly influenced chert selection.

The drainage channels incising the Nejd plateau formed during wet climatic regimes throughout the Quaternary [34]. While much of Arabia presently experiences arid/hyperarid conditions, the palaeoenvironmental record confirms that the periodic northward migration of the Inter Tropical Convergence Zone, and associated IOM rainfall, brought greater volumes of precipitation to much of the Arabian subcontinent, in particular to Dhofar. Terrestrial evidence for such pluvial episodes is found throughout Arabia within fluvio-lacustrine archives [36]–[41], speleothems [42]–[50], and deep sea cores from the Arabian Sea [51]–[54].

These data indicate that the monsoon increased in intensity during three intervals within MIS 5. Among these humid episodes, the last interglacial (sub-stage 5e; 128–120 ka) appears to represent the most significant wet phase within the entire Late Pleistocene, with rainfall surpassing all subsequent pluvials [42], [43]. Later, less substantial humid episodes associated with sub-stages 5c (110–100 ka) and 5a (90–74 ka) are also attested to in the palaeoenvironmental record. Uncertainties remain concerning the extent to which the climate deteriorated in the intervening sub-stages 5d (120–110 ka) and 5b (100–90 ka). Speleothem records indicate a change in isotope ratios and a hiatus in formation during these phases [42], however, high-resolution terrestrial data are sparse given the limited preservation of sediment during phases of aridity. It is likely that regional orographic controls on precipitation played a significant role during these dry episodes, enabling certain zones favored with topographic relief to receive some degree of consistent rainfall throughout MIS 5 (i.e., the Yemeni highlands and Dhofar).

Climate records indicate that MIS 4 (74–60 ka) was a period of rapid global cooling, at which time much of Arabia was beset by prolonged aridity caused by the southward displacement of the IOM. Records from the Arabian Sea attest to a period characterized by cooler sea surface temperatures, low productivity, and increased terrigenous (aeolian) input [56]–[58]. Studies of dune formation in the Wahiba desert [39], [55] also provide evidence of widespread desiccation indicated by aeolian accumulation throughout MIS 4, while speleothem records from Oman [42], [46], [47] record no growth during MIS 4.

Activation of Arabian fluvial systems during humid phases eg., [59], would have provided a significant source of fresh water; consequently, an increase in vegetation cover and the expansion of certain fauna. Such pluvial events are thought to have facilitated the exchange of plant and animal species between Africa and southern Arabia. The flora of Dhofar is composed of East African-derived species such as Acacia sp. (Acacia), *Ziziphus ziziphus* (Jujube), *Adansonia digitata* (Baobob), Ficus sp. (Figs), *Calotropis procera* (Sodom's Apple), and *Adenium obesum* (Desert Rose) [34], [35], [60]. While terrestrial snails found in northern Oman are primarily Palaearctic (Eurasian) taxa, the snails of Dhofar are a species rooted in East Africa [61]. Fernandes et al. [62] report mitochondrial DNA (mtDNA) evidence for a recent genetic divergence between African and Arabian genets. They list several other small and medium-sized carnivores, including the mongoose, desert fox, honey badger, caracal, jungle cat, and golden jackal that occur in both South Arabia and East Africa, which may also share a recent common ancestor. Genetic analyses of African and Arabian Hamadryas baboon populations show multiple range expansions from MIS 7 to MIS 5 [63]. There is genetic evidence for extant human population movement across the southern Red Sea, corresponding to the Holocene climatic optimum [64]. Given this exchange of African and South Arabian flora and fauna, particularly during humid episodes, it logically follows, *a fortiori*, that the archaeological record will demonstrate cultural affinities at such times.

## Results

**Figure 4:**
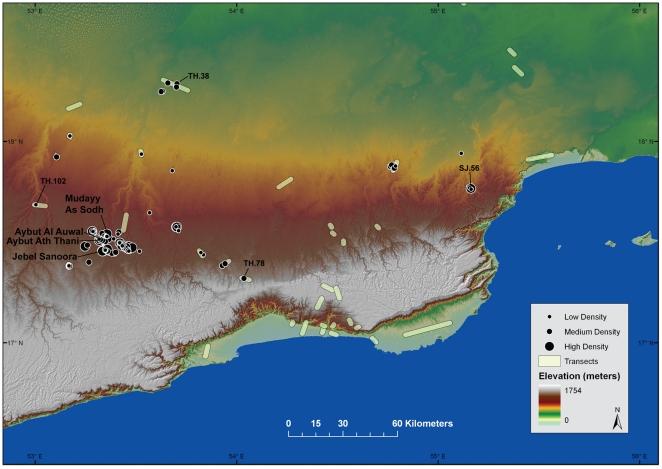
Digital elevation model of Dhofar and Nubian Complex site distribution. Survey transects covered during the 2010 and 2011 fieldwork campaigns, distribution of Nubian Complex occurrences ranked by artifact density, and specific sites mentioned in text are depicted.

DAP fieldwork was conducted over the course of two seasons in the winter of 2010 and 2011; required permits to carry out survey and excavation were granted by the Ministry of Heritage and Culture in Oman. To date, DAP has mapped 110 occurrences with Nubian Levallois technology across the Nejd plateau, ranging from occasional isolated cores to high-density scatters (Fig. 4). Lithic assemblages were collected from four of these sites to describe Nubian Levallois reduction strategies in Dhofar and to assess whether these Arabian assemblages represent a regional manifestation of the African Nubian Complex. These assemblages include: Aybut Al Auwal, Aybut Ath Thani, Mudayy As Sodh, and Jebel Sanoora. Results of the settlement survey and lithic analyses are presented below, followed by a comparison of African and Dhofar Nubian Levallois technological and typological characteristics.

### Site Distribution

Surveys were conducted along 40 transects throughout the Nejd plateau, Jebel Qara escarpment, and Salalah coastal plain (Fig. 4). Transects, ranging from two to 10 km in length, were walked by surveyors spaced roughly 10 m apart. In most cases, transects ran perpendicular to river channels to test models of site distance decay in relation to the availability of freshwater. Locations were chosen to sample the full range of geomorphic and ecological zones throughout Dhofar. Given the extensive deflationary landscapes that characterize the survey areas, there was maximum archaeological visibility along each transect. Since preservation is more or less equal across the landscape, the absence of sites can reasonably be interpreted as evidence of absence.

From the distribution of findspots in Dhofar exhibiting Nubian Levallois technology, it appears that occurrences are confined exclusively to the Nejd plateau, where they are most often found near stream channels and raw material outcrops. Survey transects did not produce evidence for any kind of MSA/MP occupation along the coastal plain or the fringes of the Jiddat Al Harassis gravel plain bordering the eastern Nejd. The westernmost occurrence (TH.102) was an isolated Nubian Type 1 core in Wadi Tanfarut along the Yemeni border, while the easternmost site (SJ.56) was a low density Nubian Levallois scatter in Wadi Qaharir, 250 km to the east. In the north, a small number of Nubian Type 1 and Type 2 cores were discovered around Shisur Farms (TH.38), on an ancient fluvial terrace 7 km east of Wadi Ghadun. Given the logistical difficulties of survey within the Rub' al Khali desert, we were not able to investigate this zone and cannot yet address the northern distribution of such sites in Dhofar. The southernmost occurrence (TH.78) was an isolated Nubian Type 1 core on a low terrace above Wadi Nirin, less than 2 km from the northern slopes of Jebel Qara. In every assemblage encountered, Nubian Type 1 cores were by far the most prevalent, and Nubian Levallois technology was never found in conjunction with a bifacial component.

Of the MSA sites with Nubian Levallois technology mapped by DAP, 39 findspots (<1 artifact per sq m), 55 low density scatters (1–10 artifacts per sq m), and 16 high density scatters (>10 artifacts per sq m) were recorded. While isolated findspots and low density scatters are found across the entire plateau, evidence for intensive/recurrent settlement is concentrated in the west-central Nejd, around a large catchment system made up of Wadis Aybut, Banut, Amut, and Ghadun. This may be linked to the presence of ancient and modern groundwater-fed springs that emerge around the village of Mudayy, at the confluence of Aybut and Banut. Not only would this zone have provided a considerable amount of water in both its rivers and springs, but also fluvial downcutting would have continually excavated fresh *Mudayy* member chert beds as the channels developed. The Aybut-Banut-Amut-Ghadun drainage system is unlikely, however, to be the only center of MSA occupation on the Nejd. We have systematically surveyed less than 1% of the 33,000 km^2^ plateau, so it is likely that there are other catchments with similarly high concentrations of MSA artifacts.

### Aybut Al Auwal

**Figure 5:**
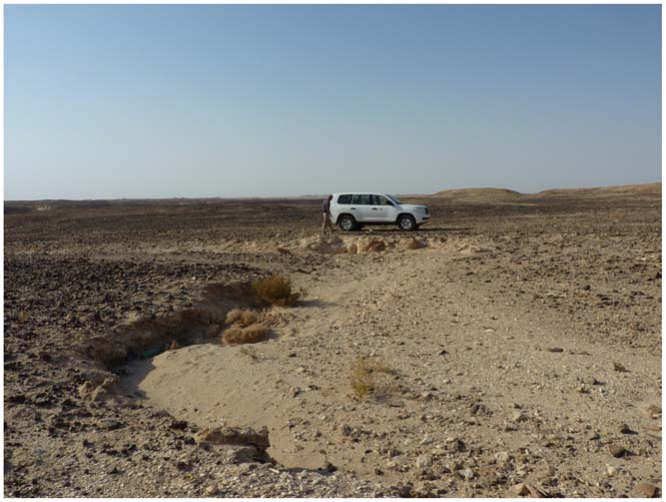
Photo of Aybut Al Auwal gully. One of the meandering stream channels incising the chert-covered terrace. Excavation section is immediately in front of car.

**Figure 6:**
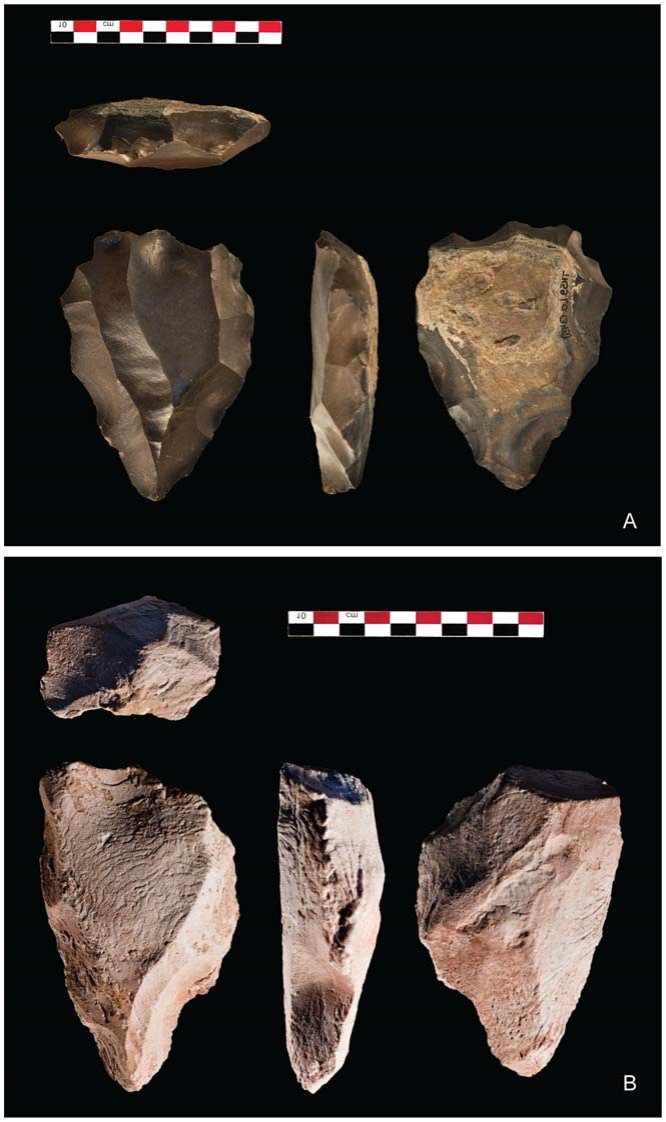
Nubian Type 1 cores from Aybut Al Auwal. Core in panel A shows dark patination/varnish and was collected from terrace surface, while core depicted in panel B is partially desilicified and was excavated from stratigraphic Unit 3.

Aybut Al Auwal (“First Aybut”) is an open-air site that contains artifacts on the surface and buried within fluvial sediments in Wadi Aybut, west-central Nejd. The site was found on the second terrace, ∼20 m above a relict tributary channel feeding the main wadi system. The terrace is blanketed in a pavement of naturally occurring *Mudayy* chert and chipping debris, and is incised by a series of small stream channels (Fig. 5). Lithic artifacts were found cemented within and eroding from accretional sediments filling the channel. Both natural and archaeological surface debris are coated in a black desert varnish (Fig. 6A), while the buried material is bleached white and partially desilicified from chemical dissolution (Fig. 6B). Although the artifacts do not have edge damage from post-depositional movement, many of the pieces exhibit rounded ridges from wind abrasion and surface water runoff across the terrace.

The Aybut Al Auwal terrace is formed by unconformities within horizontal strata of the underlying bedded chert (*Mudayy* member) and Tertiary limestone (*Umm Ar Radhuma* formation) [34]. Two small (<3 m wide) westward-flowing streams incise the terrace and debouch over a knickpoint that forms a water drop onto a lower terrace, feeding the upper tributaries of the nearby Wadi Aybut. Stream migration on the Aybut Al Auwal terrace is largely controlled by variations in the morphology of the underlying bedrock and surface density of the overlying exposed chert beds, which may also have been anthropogenically displaced by chert exploitation. Stream channels incise the terrace to a maximum depth of ∼1 m, and the lateral accretion of sediments due to channel migration occurred at two sharp meanders within these channels.

The stream at Aybut Al Auwal has undergone at least one phase of channel incision followed by the lateral accretion of sediments during stages of channel migration. Sediment preservation is minimal, however, given the relatively small size of the channels and their close proximity to the local watershed. The now-relict channels are easily identifiable within the landscape due to partial infilling with pale, calcareous fines and an absence of large (i.e. >10 cm) limestone and chert clasts within their course.

**Figure 7:**
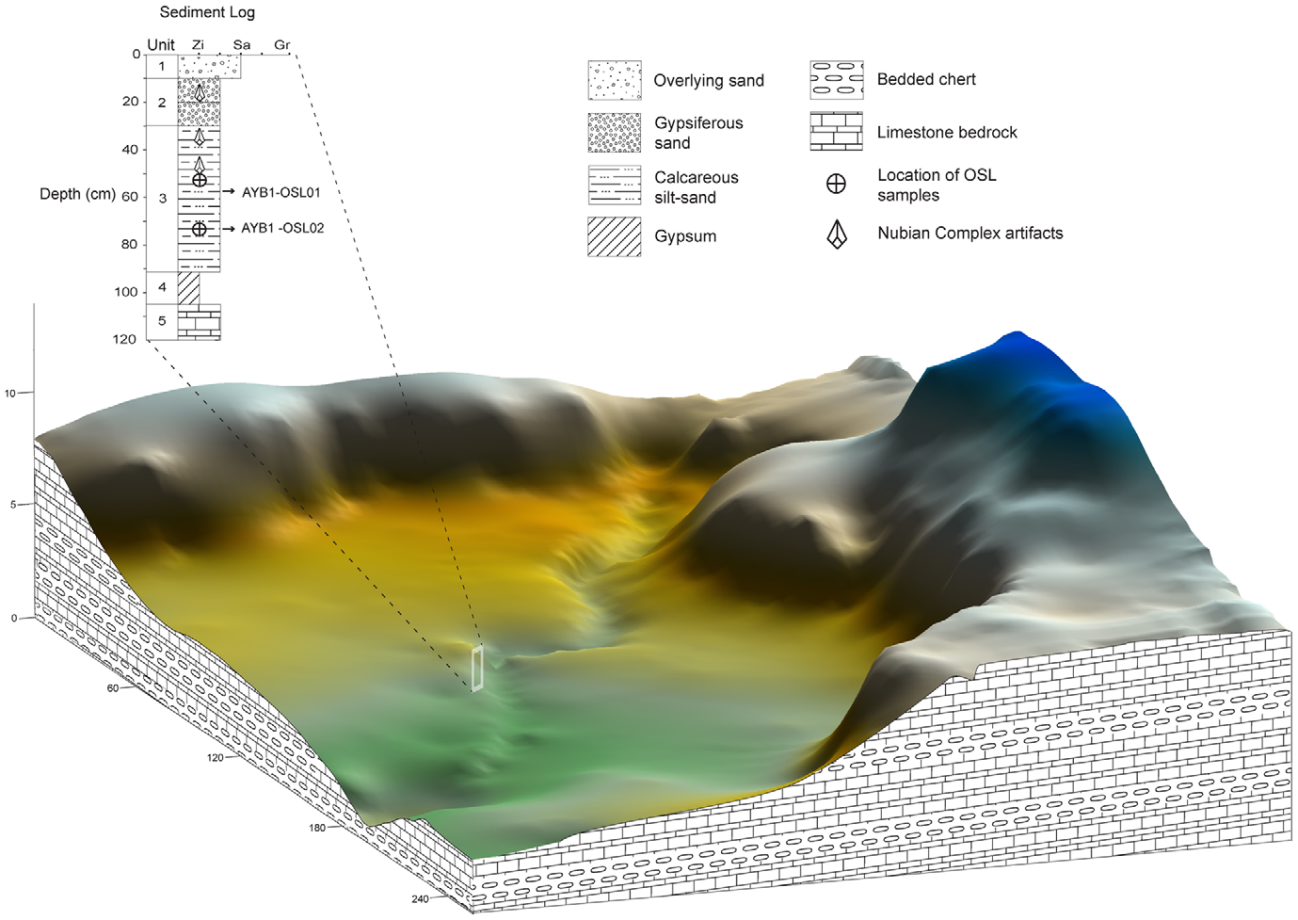
Topographic relief of Aybut Al Auwal terrace (vertically exaggerated) and sediment log.

One such lateral channel-fill deposit was excavated to a depth of 92 cm and is comprised of four distinct stratigraphic units, which overlie the limestone channel bed (Fig. 7). The uppermost unit, Unit 1, is capped by worked and unworked chert clasts at the surface and is comprised of non-laminated, homogeneous pale-brown sand that likely reflects a deflationary surface. The underlying Unit 2 consists of loosely-cemented, gypsiferous (granular) silt-sand sediment with no distinct bedding structures. An abrupt facies change at a depth of ∼30 cm marks the transition to Unit 3, which is a highly cemented sedimentary stratum composed of homogeneous white, fine-grained, calcareous silt-sized material with only a minimal sand-sized component. This unit represents the lateral accretion of suspended fluvial sediments that have been eroded from the surrounding bedrock and deposited downstream, along with lithic artifacts and chert debris that slumped in from the surface as the terrace was undercut. As there is no sedimentary evidence of a hiatus in deposition throughout Unit 3, it appears that stream flow was relatively uninterrupted and represents a single phase of deposition. A well-developed gypsum layer, Unit 4, is sharply bounded by both the overlying fluvial sediments of Unit 3 and by the underlying limestone bedrock.

**Figure 8:**
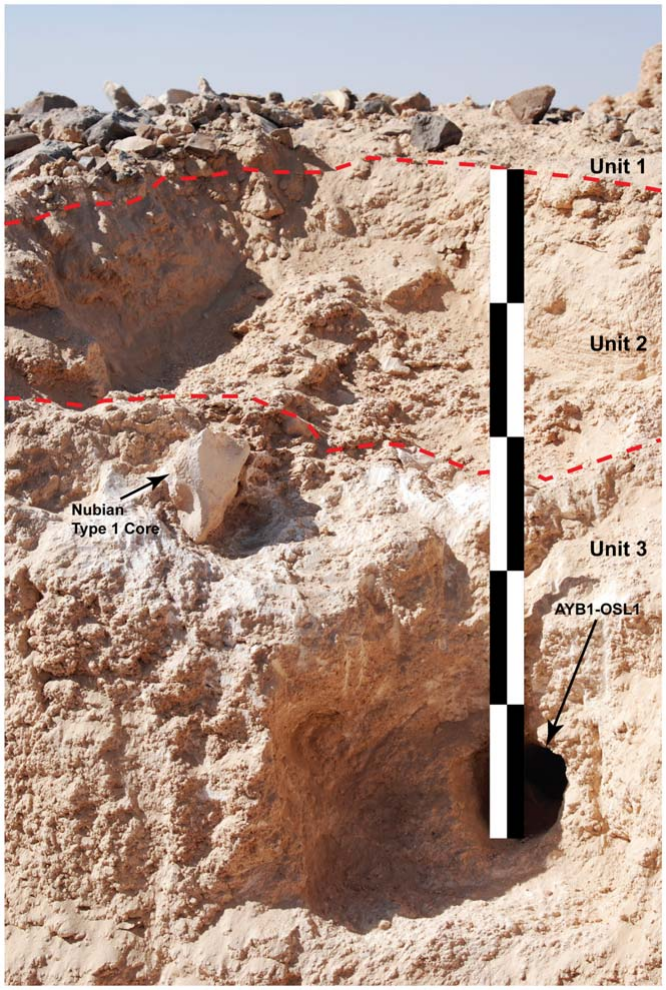
Photo of buried Nubian Type 1 core in situ. Position of artifact is shown in relation to AYB1-OSL1 sample; both are within stratigraphic Unit 3.

The depositional age of the artifact-bearing sediments in Unit 3 was estimated by OSL dating of buried quartz grains [65] collected from depths of ∼52 cm (sample AYB1-OSL1: 106±9 ka) and ∼74 cm (sample AYB1-OSL2: 107±9 ka). The OSL ages for these two samples are statistically concordant (Table S1) and give a weighted mean age of 106.6±6.4 ka for the accretion of Unit 3 fluvial sediments (see Appendix S1 and Figure S1 for details of OSL dating methods and results). This reflects the elapsed time since the dated quartz grains were last exposed to sunlight, and indicates that the stream channel at Aybut Al Auwal was active during MIS 5c. Within Unit 3, there are no bedding structures or facies changes to indicate lacunae of deposition, corroborating the coeval OSL estimates. It is a homogenous layer that accumulated during a single, continuous phase of deposition. There were two technologically diagnostic artifacts from Unit 3, including a Nubian Type 1 core (Fig. 6B) found just above the OSL sample AYB1-OSL1 (Figure 8), and the proximal-medial fragment of a Levallois point with chapeau de gendarme striking platform and converging lateral edges. Despite being somewhat desilicified, the buried artifacts are in good condition and diagnostic of Nubian Type 1 technology. As the OSL measurements and sedimentology indicate that all of Unit 3 formed during one accretional episode, we conclude that the buried Nubian artifacts were deposited ∼106 ka, when the channel was active. Albeit slightly earlier than its African counterpart, the age of the Aybut Al Auwal assemblage is more or less consistent with the numerical ages obtained from the Nile Valley [21], Red Sea hills [10], and eastern Sahara [7], [8] (Table 1).

**Figure 9:**
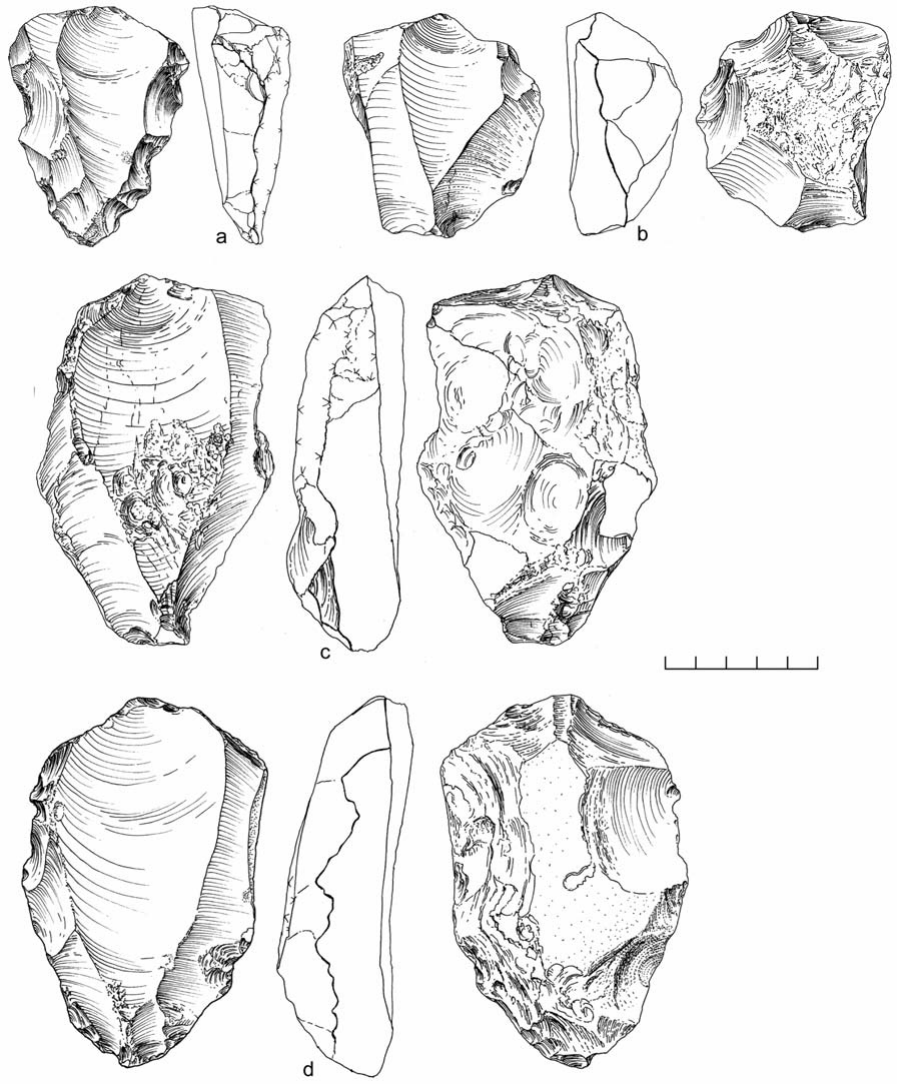
Nubian Levallois cores from Aybut Al Auwal. Type 1 (b,c,d) and Type 2 (a).

**Figure 10:**
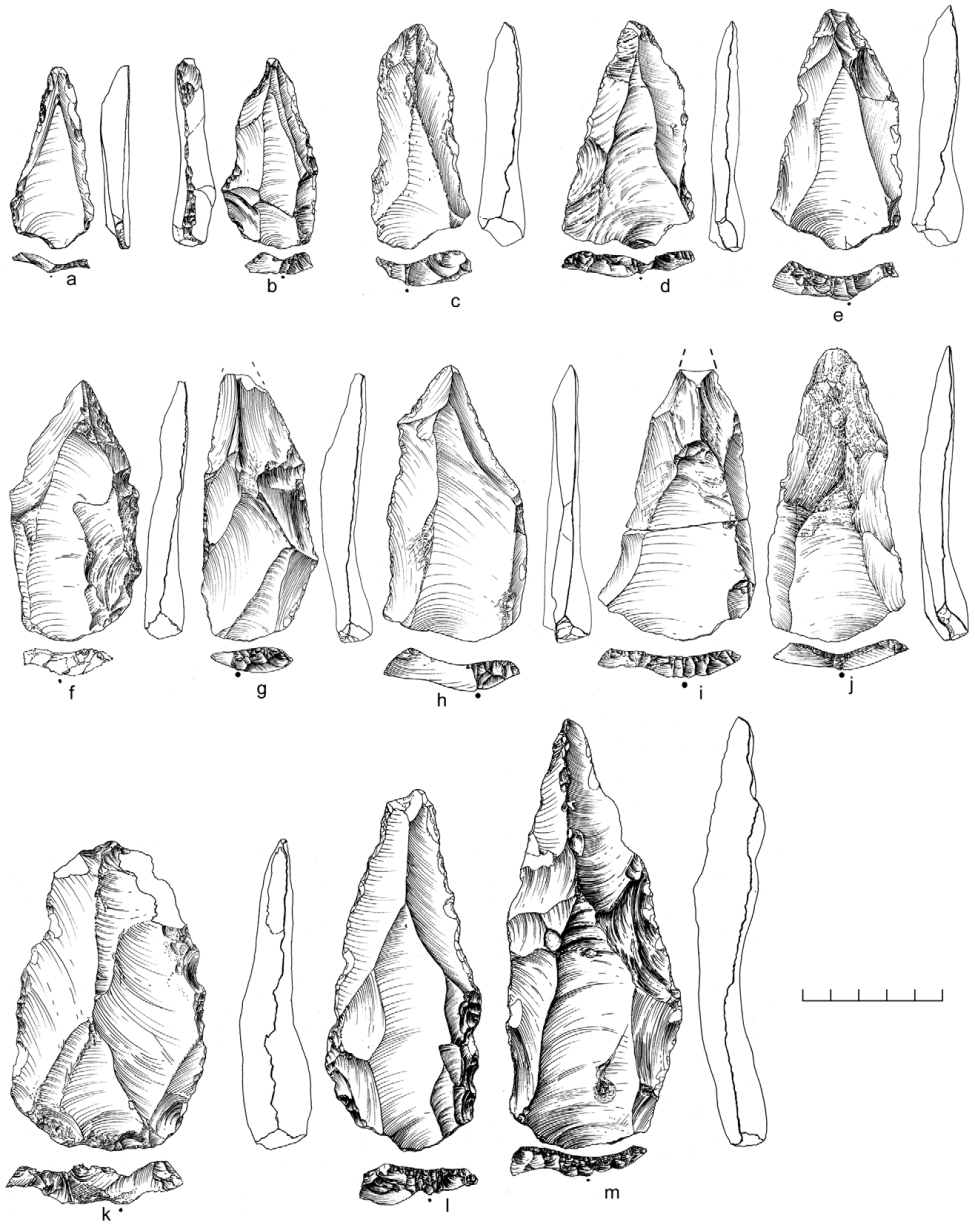
Levallois points from Dhofar Nubian Complex sites. Aybut Al Auwal (c,e,f,k), Aybut Ath Thani (a,b), Mudayy As Sodh (i), Jebel Sanoora (j), TH.173 (d), TH.236 (m), TH.238 (g,h), and TH.258 (h).

A random collection of surface material from the terrace recovered 859 artifacts from ∼2,500 m^2^. An additional 10 pieces were excavated from ∼1 m^3^ of highly-cemented sediment comprising stratigraphic Unit 3, and 11 desilicified artifacts were collected nearby eroding from the side of the channel (Table 2). Both the surface and buried assemblages are characterized almost exclusively by Nubian Levallois technology, with 79% of cores classified as Nubian Levallois (Table 3; Fig. 9). Of these, Nubian Type 1 account for nearly 60% of all cores, while less than 10% are Nubian Type 2 (Table 3). Accompanying the Nubian cores, a large number of Levallois flakes, blades, and points were identified with faceted, dihedral, and *chapeau de gendarme* striking platforms (Fig. 10C, 10E, 10F, 10K). Debordant blades, a byproduct of Levallois primary working surface preparation, are among the most frequent blank types (Table 4).

**Table 2:** Artifact class by site.

	Aybut Al Auwal^1^	Aybut Ath Thani	Mudayy As Sodh	Jebel Sanoora
Debitage	407	1503 (86.7)	804 (83.3)	330 (73.5)
Cores	297	157 (9.1)	92 (9.5)	104 (23.2)
Tools	176	74 (4.3)	69 (7.2)	15 (3.3)
Total	880	1734	965	449

1 Percentages and technological indices omitted from Aybut Al Auwal given the non-systematic collection.

**Table 3:** Core types by site.

	Aybut Al Auwal^1^	Aybut Ath Thani	Mudayy As Sodh	Jebel Sanoora
Nubian Type 1	176	111 (70.7)	44 (47.8)	28 (26.9)
Nubian Type 2	19	12 (7.6)	9 (9.8)	16 (15.4)
Nubian, indeterminate	40	16 (10.2)	19 (20.7)	25 (24.0)
Centripetal Levallois	19	2 (1.3)	2 (2.2)	0
Radial	3	5 (3.2)	0	0
Bidirectional	9	3 (1.9)	2 (2.2)	8 (7.7)
Convergent	7	0	0	2 (1.9)
Single platform	12	6 (3.8)	10 (10.9)	19 (18.3)
Opposed platform	1	1 (0.6)	2 (2.2)	1 (1.0)
Crossed	2	1 (0.6)	2 (2.2)	3 (2.9)
Orthogonal	1	0	2 (2.2)	2 (1.9)
Pre-core	8	0	0	0
Total	297	157	92	104

1 Percentages and technological indices omitted from Aybut Al Auwal given the non-systematic collection.

**Table 4:** Debitage cortex % and technological indices by site.

	Aybut Al Auwal^1^	Aybut Ath Thani	Mudahh As Sodh	Jebel Sanoora
Flakes (no cortex)	97	197 (37.5)	242 (30.1)	127 (38.5)
Flakes (1–50% cortex)	141	103 (19.6)	254 (31.6)	45 (13.6)
Flakes (51–100% cortex)	65	161 (30.6)	182 (22.6)	34 (10.3)
Blades (no cortex)	39	23 (4.4)	40 (5.0)	28 (8.5)
Blades (1–50% cortex)	48	18 (3.4)	67 (8.3)	67 (20.3)
Blades (51–100%)	17	24 (4.6)	19 (2.4)	29 (8.8)
Total	407	526	804	330
Blade index	N/A^1^	12.4	15.7	37.6
Levallois index	N/A^1^	7.4	4.6	3.0

1 Percentages and technological indices omitted from Aybut Al Auwal given the non-systematic collection.

Tools are numerous (Table 2), accounting for 20% of the total assemblage. This unusually high frequency is partially due to non-systematic collection bias. Tools include standard MSA types such as Levallois points, Levallois flakes/blades, sidescrapers, endscrapers, denticulates, notches, perforators, and retouched pieces (Table 5). The sole burin within the assemblage was on a truncation, struck from an abruptly retouched edge. Nearly all of the endscrapers are nosed. Bifacial foliates, which are common among early Nubian Complex sites in Africa, are absent at Aybut Al Auwal. Considering the significantly greater number of Nubian Type 1 over Nubian Type 2 cores, as well as the complete lack of bifacial reduction, the Aybut Al Auwal assemblage resembles the late Nubian Complex of northeast Africa.

**Table 5:** Tool types by site.

	Aybut Al Auwal^1^	Aybut Ath Thani	Mudayy As Sodh	Jebel Sanoora
Levallois points^2^	60	21 (28.4)	18 (26.1)	5 (33.3)
Levallois flakes/blades^2^	55	18 (24.3)	19 (27.5)	5 (33.3)
Sidescrapers	23	34 (45.9)	7 (10.1)	2 (13.3)
Endscrapers	13	0	7 (10.1)	0
Denticulates	2	0	7 (10.1)	1
Notches	11	0	4 (5.8)	0
Burins	1	1 (1.4)	0	0
Perforators	5	0	0	0
Retouched pieces	6	0	7 (10.1)	2 (13.3)
Total	176	74	69	15

1 Percentages and technological indices omitted from Aybut Al Auwal given the non-systematic collection.

2 For the purposes of this typological analysis, all Levallois end products are classified as tools. This is to maintain consistency with the Bordian classification system and to enable comparisons with other Nubian Complex publications.

### Aybut Ath Thani

**Figure 11:**
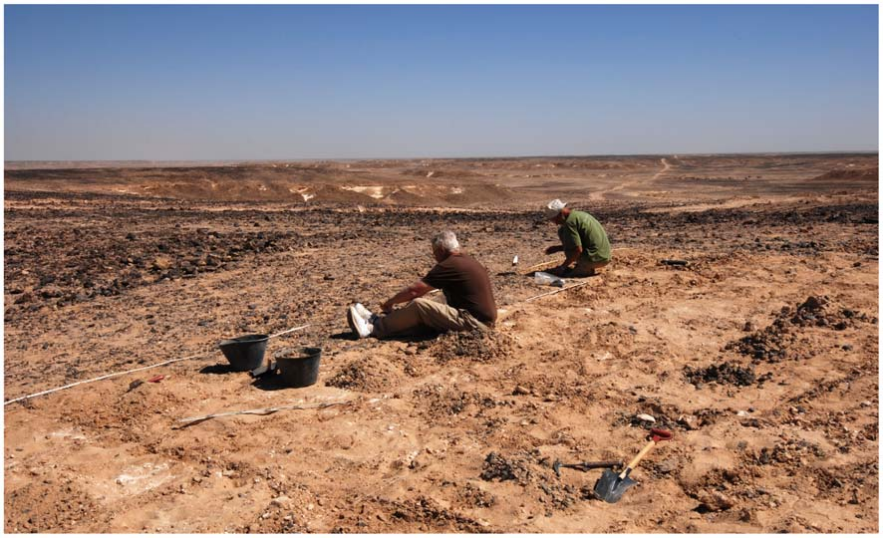
Photo of Aybut Ath Thani. DAP team systematically collects surface material from gridded area with view overlooking Wadi Aybut in background.

Aybut Ath Thani (“Second Aybut”) is a Nubian Complex surface scatter situated on a gravel plain some 5 km northeast of Aybut Al Auwal. The site is positioned at the headwaters of two large tributary systems, with prominent views of wadi channels to the east and west (Fig. 11). Although there is adequate *Mudayy* chert outcropping within ∼250 m, there is no raw material source directly at the site.

The small lithic scatter observed at Aybut Ath Thani is constrained to no more than 400 m^2^. A 10×10 m area was systematically collected in 1 m^2^ units, and all cores, tools, and a 25% sample of debitage were analyzed. Cores and larger pieces of debitage are only moderately weathered, however, the smaller material is in exceptionally poor condition, due to a combination of taphonomic processes including deflation, winnowing, surface runoff, chemical alteration, and thermal fracturing. While striking platforms and scar patterns are clear and permit technological analysis, the resulting edge damage caused by these destructive processes has obscured possible retouch, hindering typological identification. Given this problem, the Aybut Ath Thani tool type list should be approached with caution.

**Figure 12:**
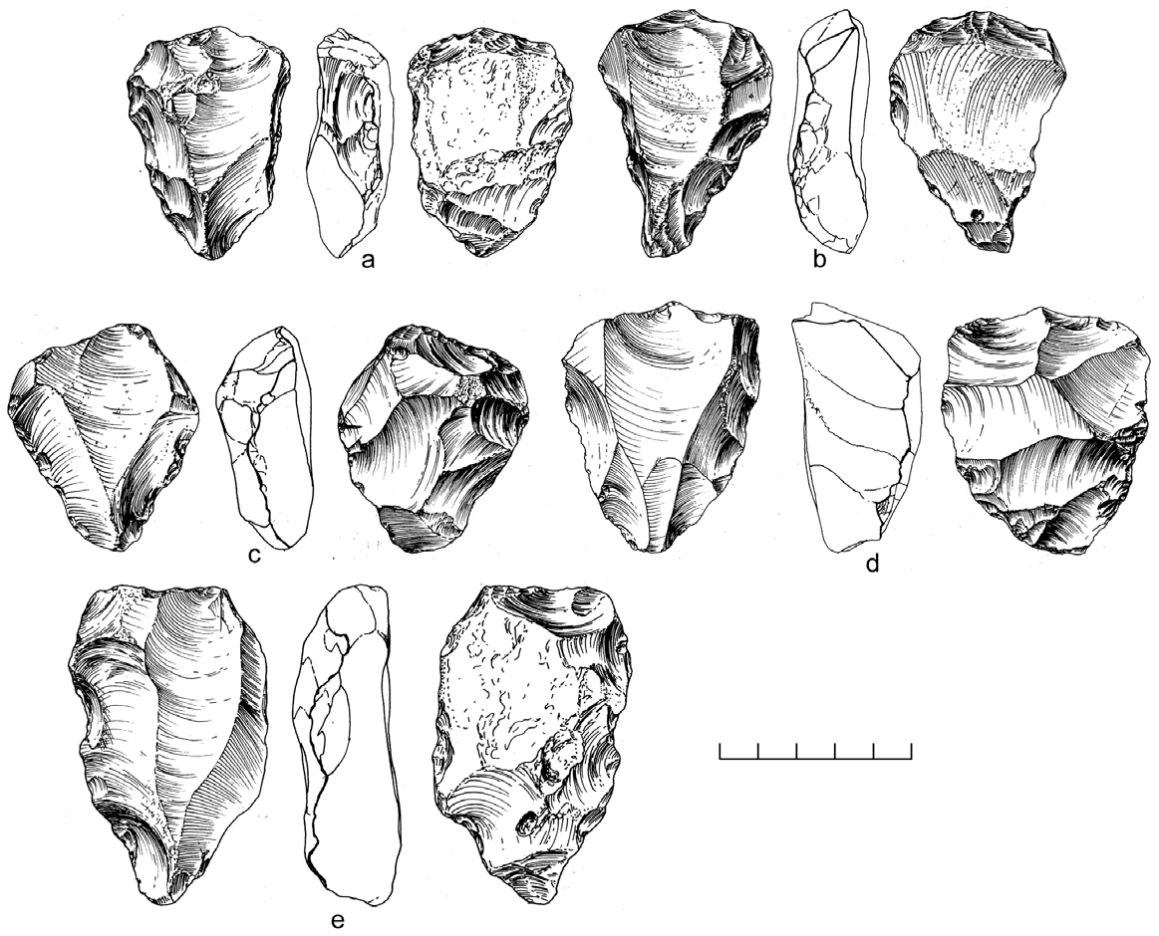
Nubian Levallois cores from Aybut Ath Thani. Type 1 (a,c,d,e) and Type 2 (b).

Of the 1,734 artifacts comprising the Aybut Ath Thani assemblage, 157 (9%) are cores (Table 2). Nubian Levallois accounts for a higher proportion of core types (almost 90%) than in any of the other Dhofar assemblages (Table 3; Fig. 12). Several of the Nubian cores were broadly identified as such, but could not be placed within a specific category because they were either in early stages of preparation or the preferential blank was overpassed, removing the signature distal ridge on the primary working surface. Single platform, radial, bidirectional, and non-Nubian Levallois constitute just over 10% of all other core types.

Although the site is positioned slightly away from a source of raw material, there is a relatively low ratio of non-cortical pieces to cortical pieces (Table 4). There are more primary blanks than at the other sites examined in this study, which are all located directly on raw material sources. This trend suggests that unmodified nodules were brought to Aybut Ath Thani and the primary stage of reduction was carried out on site.

**Figure 13:**
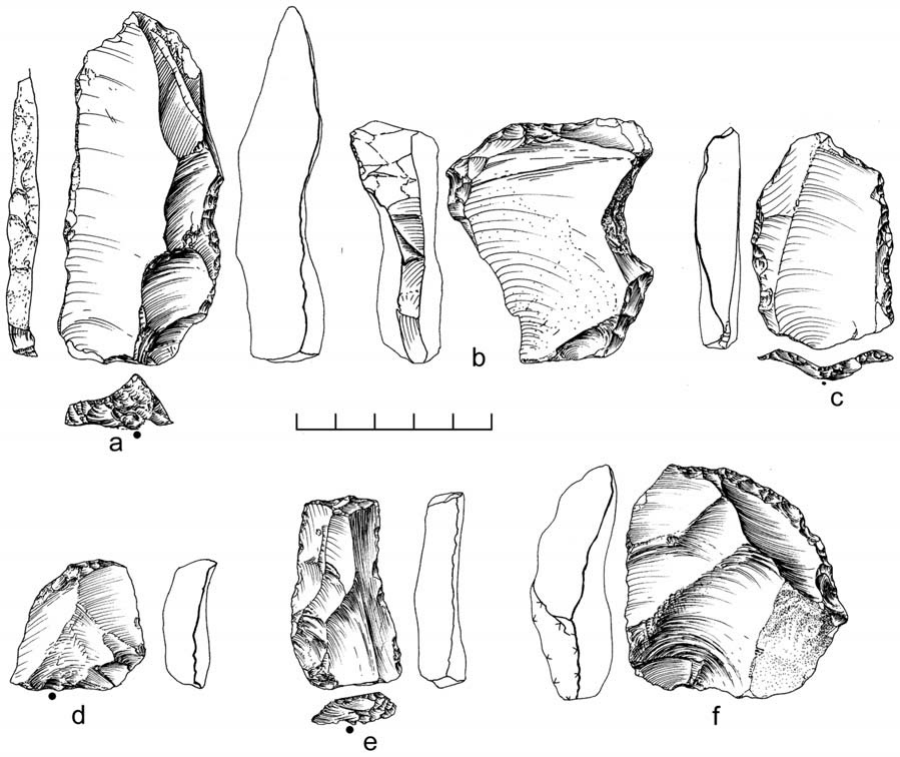
Retouched tools from Dhofar Nubian Complex sites. Sidescrapers from Aybut Ath Thani (c) and Mudayy As Sodh (f), endscrapers from Mudayy As Sodh (b,d,e), and notch from Mudayy As Sodh (a).

Some blanks were identified with sufficiently consistent retouch to be classified as tools, despite the heavy edge damage on many of the pieces in this assemblage. These types, presented in Table 5, include sidescrapers (Fig. 13C), Levallois points (Fig. 10A–B), Levallois flakes and blades, and a single burin. It is likely that this lack of variability in tools is due to the destructive taphonomic processes noted above, skewing the sample toward the most easily recognizable types. The absence of bifacial technology, along with a much higher frequency of Nubian Type 1 to Type 2 cores, again, is indicative of the late Nubian Complex.

### Mudayy As Sodh

Mudayy As Sodh (“Mudayy's Rooftop”) is located on a high plateau above the village of Mudayy. The site consists of multiple surface scatters just over 1 km east of Aybut Al Auwal, around a series of shallow basins that debouch into the main Aybut tributary. Small gullies (<50 cm deep) incise the silicate gravel covering the plateau, where a variety of assemblages were observed in discrete patches across the landscape. Nubian Complex scatters were identified closer to the edge of the plateau overlooking the drainage systems below, while less weathered Nejd Leptolithic [66], [67] concentrations were observed at the base of the low hills on the plateau, associated with more recently exposed chert beds. The extent and density of Nubian Complex scatters at the Mudayy As Sodh locality are probably linked to an earlier phase of erosion that exposed high-quality *Mudayy* member chert beds, as the soft limestone hills were broken down by wind and surface runoff.

**Figure 14:**
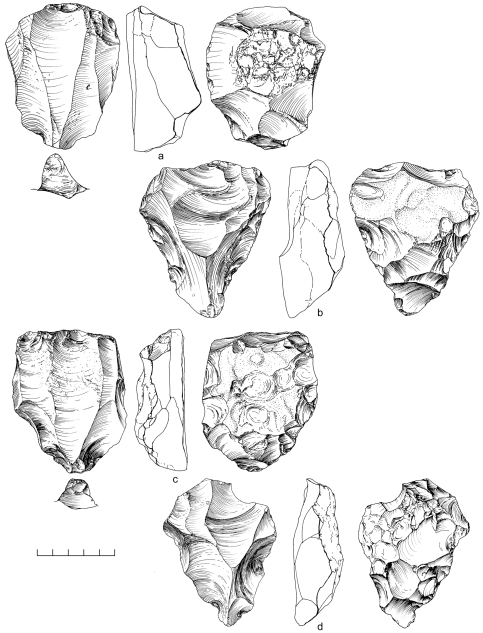
Nubian Levallois cores from Mudayy As Sodh. Type 1 (a,c,d) and Type 2 (b).

**Figure 15:**
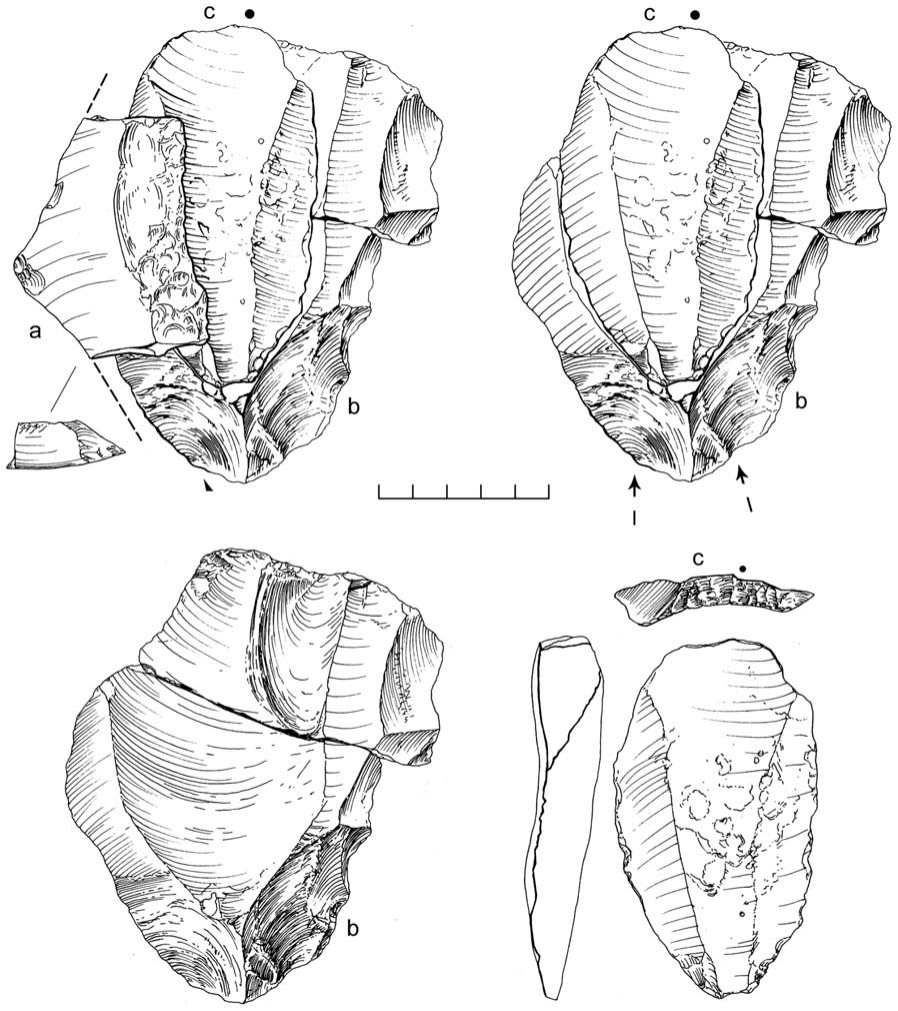
Nubian Levallois refit from Mudayy As Sodh. Levallois point (c) and debordant blade (a) conjoin with Nubian Type 1 core (b).

**Figure 16:**
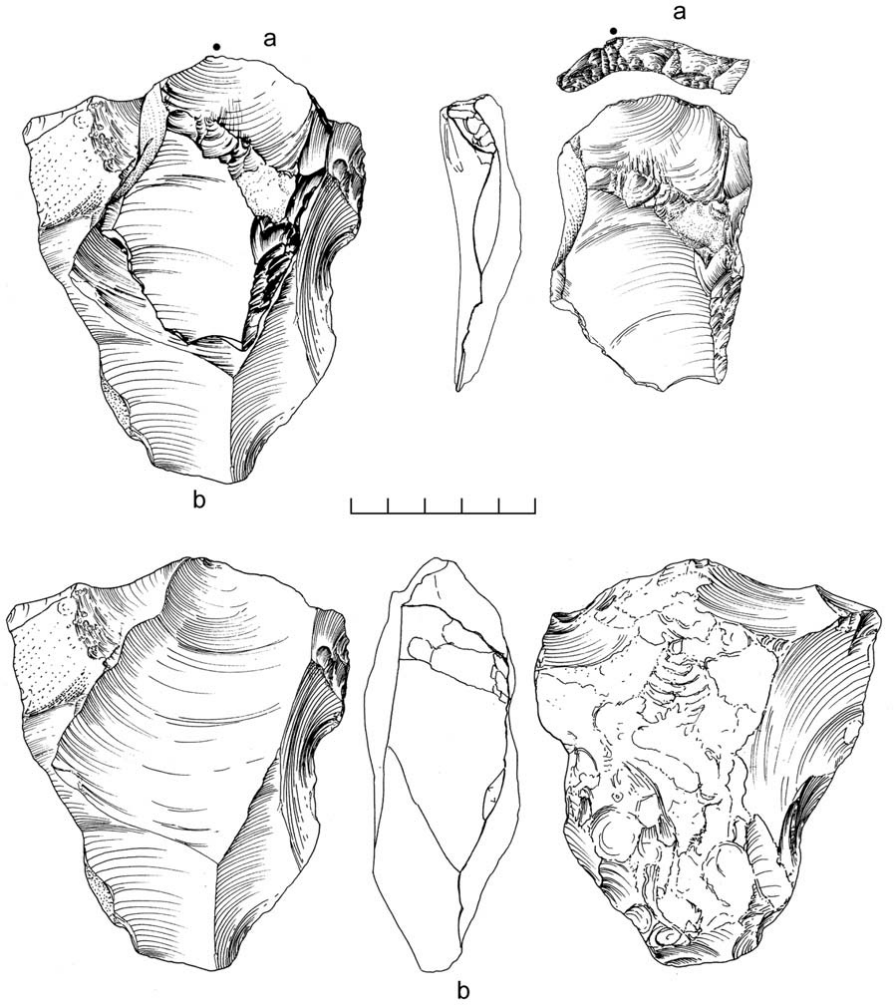
Nubian Levallois refit from Mudayy As Sodh. Levallois point (a) conjoins with Nubian Type 1 core (b).

An area of 64 m^2^ was systematically collected from one Nubian concentration at Mudayy As Sodh, chosen for its high density of cores and debitage. 965 artifacts were recovered in total, including 92 cores, 69 tools, and 804 pieces of debitage (Table 2). Nubian cores were the most prevalent, accounting for 78% of all variants, of which most were Type 1 (Fig. 14). Nubian core conjoins within the assemblage attest to minimal post-depositional disturbance of the scatter (Figs. 15, 16). Occasional single platform, bidirectional, opposed platform, and orthogonal cores occur in low percentages (Table 3).

Tools make up just over 7% of the Mudayy As Sodh assemblage. Over half of the toolkit is comprised of Levallois points (Fig. 10I), flakes, and blades, while remaining types include sidescrapers (Fig. 13F), endscrapers (Fig. 13B, 13D, 13E), denticulates, notches (Fig. 13A), and retouched pieces (Table 5). Like most other MSA sites in Dhofar, there is a predominance of Nubian Type 1 cores and no bifacial component, indicative of the late Nubian Complex industry.

### Jebel Sanoora

**Figure 17:**
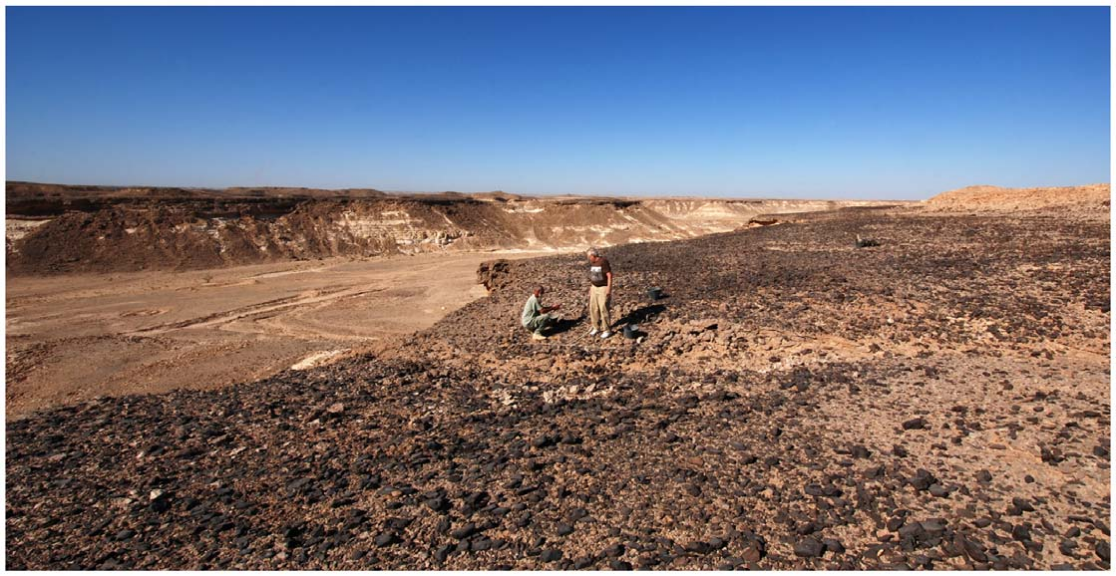
Photo of Jebel Sanoora terrace. DAP team systematically collects surface material from gridded area at edge of terrace. Terrace shows dense chert cover of natural and worked debris.

Jebel Sanoora (“Cat Hill”) is situated 6 km southeast of Aybut Al Auwal. The site consists of several concentrated lithic scatters on the first erosional terrace (about 5–15 m wide), perched ∼10 m above a steeply-incised wadi channel (Fig. 17). Exceptionally high-quality chert slabs of the *Mudayy* geological member outcrop across the entire terrace, with more heavily weathered chert nodules found closer to the edge of the terrace, and less weathered raw material actively outcropping from the base of the higher terrace. As was the case at Mudayy As Sodh, Nejd Leptolithic assemblages were found in association with fresh outcrops, while older Nubian artifacts were noted only along the edge of the terrace. The Nejd Leptolithic material was in pristine condition, yet many of the Nubian artifacts have undergone aggressive chemical weathering that has left their surfaces heavily discolored and pitted.

**Figure 18:**
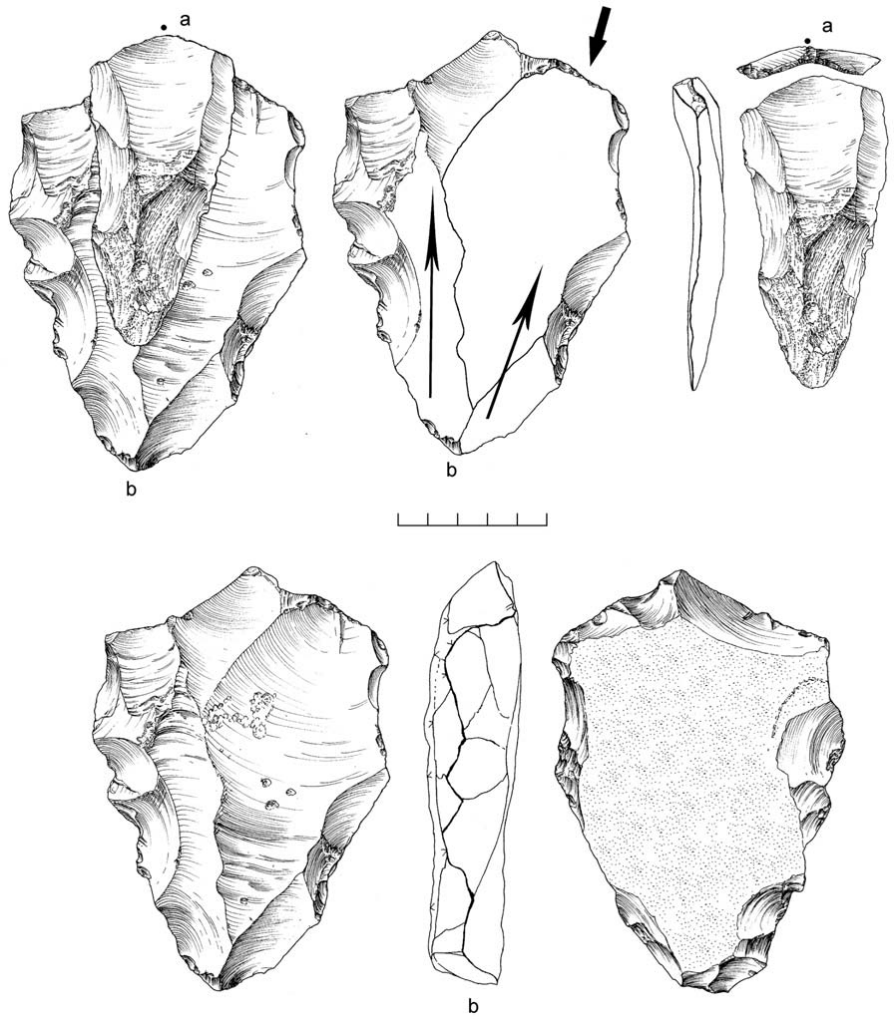
Nubian Levallois refit from Jebel Sanoora. Levallois point (a) conjoins with Nubian Type 1 core (b).

Two separate Nubian Complex scatters were systematically sampled, with a total collection area covering 20 m^2^. As there are no obvious differences between the two areas, we have combined them into a single assemblage for the purposes of this analysis. The composite assemblage is comprised of 449 artifacts; Nubian core conjoins indicate there has been minimal post-depositional disturbance (Fig. 18).

Among the cores, over half are Nubian (Table 3). While Type 1 cores are still more frequent than Type 2, the difference between the two categories is somewhat smaller than the other assemblages. The high percentage of indeterminate Nubian cores is due to a number of early-stage cores that have undergone initial Nubian distal ridge preparation, although have not been reduced enough to distinguish between Type 1 or Type 2 methods. In addition, many of the Nubian cores have overpassed primary working surfaces, obscuring evidence of distal preparation.

Single platform, bidirectional, crossed, opposed platform, orthogonal, and convergent types account for just over a third of all cores. The significant percentage of unidirectional blade cores suggests the presence of a distinct, simple blade technology within the Jebel Sanoora assemblage, also reflected in the unusually high blade index of 38% among all unmodified blanks (Table 4). Most of the blade and blade cores exhibit heavier weathering, suggesting that they may predate the Nubian component and that the Jebel Sanoora assemblage is a palimpsest of both Nubian MSA technology and an earlier laminar reduction strategy. Whether they are coeval or successive must be resolved through further investigation of the Jebel Sanoora locality.

There are just 15 tools within the assemblage, of which 10 are Levallois points, flakes, and blades. Among the remaining specimens, there are two convex sidescrapers, a denticulate, and two retouched pieces (Table 5). Again, bifacial technology is absent, signifying the late Nubian Complex industry.

### Analysis

**Figure 19:**
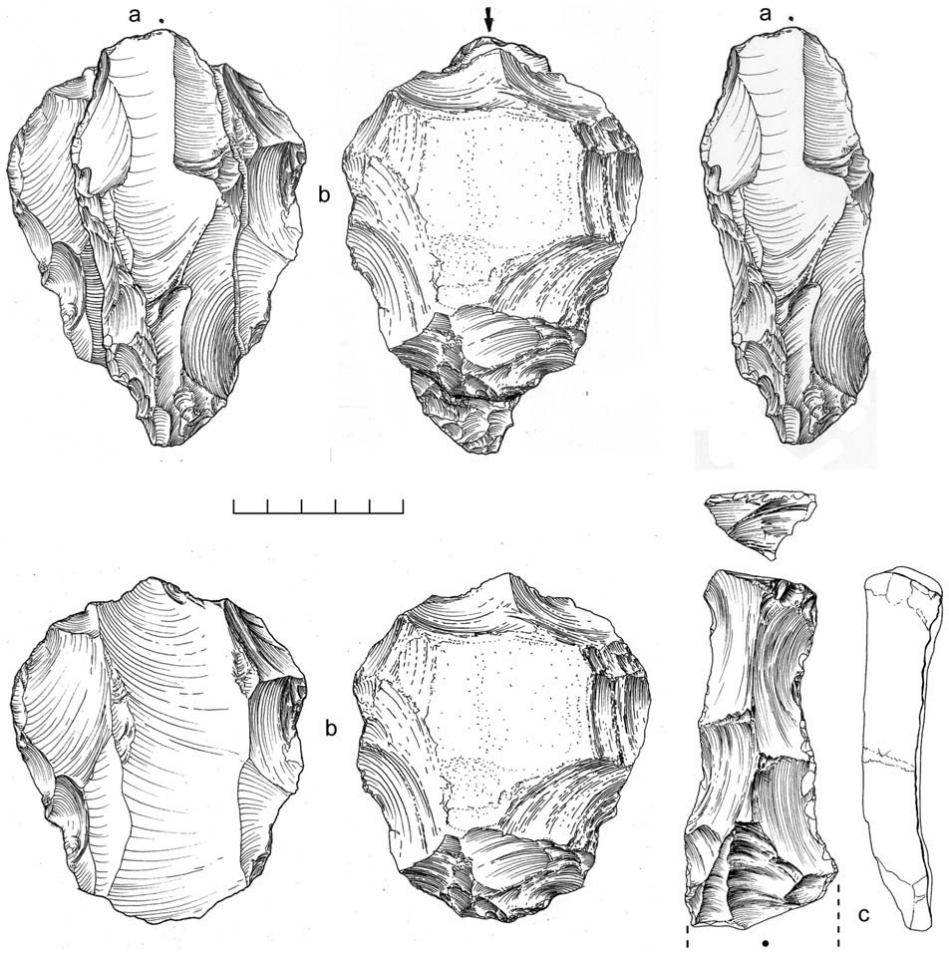
Examples of Nubian Levallois refits at Aybut Al Auwal. Overpassed Levallois blade (a) conjoins with Nubian Type 1 core (b). Distal fragment of overpassed Levallois blade (c) showing prominent distal ridge.

There are close affinities between assemblages discovered in Dhofar and the late Nubian Complex of northeast Africa. The essential feature of Nubian Levallois technology is the creation of a prominent distal median ridge formed by steeply angled distal (Type 1) and/or steep bilateral (Type 2) removals. Specimens from Dhofar exhibiting this characteristic distal median ridge are shown in cross section in Figs. 14A, 14C and 19C. African and Dhofar Nubian Complex reduction strategies, in this regard, are the same. Moreover, the Nubian Type 1 process of preparing convexity across the primary working surface of the core is mirrored in Africa and southern Arabia, to a high degree of standardization. In both regions, divergent lateral blanks were struck from the distal end of the core to set up for the preferential removal of an elongated pointed blank, in the process producing a large number of debordant blades with bidirectional scar patterns. Platform faceting is another common feature, in some cases with well-constructed chapeau de gendarme striking platforms (e.g., Fig. 10E). Given these closely overlapping characteristics, we conclude that Nubian Levallois core reduction strategies are virtually identical on both sides of the Red Sea.

Nubian Complex assemblages in northeast Africa exhibit multiple core types, including Nubian Type 1, Nubian Type 2, preferential centripetal Levallois, bidirectional, and single platform (Table 6). In Dhofar, Nubian Type 1 is the most common type, followed in smaller percentages by Nubian Type 2, preferential centripetal Levallois, bidirectional, and single platform cores. Hence, the late Nubian Complex of northeast Africa and Dhofar include the same range of variability, but Nubian Levallois technology is a considerably greater component in Dhofar. This may be partially attributed to differences in classificatory criteria, but it cannot fully explain the much higher frequency of Nubian cores in Dhofar assemblages, which range from 66% to 89% of total cores.

**Table 6:** Frequency of core types in sample African Nubian Complex assemblages.

	K'One locality 5, Ethiopia(Kurashina, 1978)	1035, Sudan(Marks, 1968)	1038, Sudan(Marks, 1968)	Abydos locality 46a, Egypt(Olszewski et al., 2010)
Nubian Levallois	19 (29.2)	49 (35.3)	32 (23.3)	33 (20.5)
Centripetal Levallois	43 (30.9)	64 (46.4)	31 (48.4)	35 (21.7)
Bidirectional	3 (2.2)	2 (1.5)	3 (4.7)	0
Other (fragments, discoids, single platform, pre-cores, multiple platform, orthogonal)	47 (33.8)	40 (28.9)	15 (23.4)	93 (57.8)
Total	139	138	64	161

The most common tool types found within Nubian Complex assemblages in Dhofar are Levallois points, flakes, and blades, which show a propensity toward elongation and converging lateral edges. The relatively few retouched tools include sidescrapers, endscrapers, denticulates, notches, and miscellaneous retouched pieces, with a trace number of burins and perforators (Table 5). This same array of MSA tool types is found within late Nubian Complex assemblages in Africa [2], [13]. In both Africa and southern Arabia, the range of tools other than Levallois products are similar and infrequent, and in both cases, the late Nubian Complex has no bifacial component.

Given these technological and typological similarities, we classify the Dhofar assemblages as late Nubian Complex. It is more likely that the high degree of overlap observed in southern Arabian and northeast African Nubian Complex assemblage – a continuous phytogeographic zone divided only by the Red Sea – is the result of cultural exchange, rather than the synchronistic result of concurrent technological evolution. For the time being, the apparent distribution of Nubian Levallois technology in Arabia is limited to the Nejd plateau and, perhaps, Hadramaut valley (Fig. 1). Archaeological surveys in central/northern Oman have not produced any evidence of Nubian Complex occupation [66], [68], nor have Nubian Complex occurrences yet been found in eastern [22], [69]–[71], central, or northern Arabia [72]–[74]. Considering the Nubian Complex occupations at Sodmein Cave in the Red Sea hills, Egypt, and the purported Nubian cores found in Sinai [27], it would not be surprising to find additional Nubian Complex occurrences within drainage systems along the western coast and hinterlands of central Arabia.

While any explanation must be speculative, we suggest that the significantly higher frequency of Nubian cores in Dhofar, as opposed to the Nile Valley, may be the result of variations in hunting behavior across the two landscapes. These differences, in turn, are the function of hydrology and the area of exploitable land in Dhofar versus the Nile Valley. In southern Arabia during MIS 5c, there were extensive grasslands cut by drainages, but none so big as to limit faunal distributions or impede hunter-gatherer mobility. In the Nile Valley, on the other hand, exploitable land was limited to the valley itself and to a narrow strip of land along its sides. Both to the east and west of the Nile Valley, the flat gravel plains would not have been appropriate hunting terrain, as confirmed by the lack of sites even a few kilometers from the valley [1], [2], [75], [76]. Thus, we propose that in Dhofar, the settlement and exploitation systems were more mobile and less compacted than those around the Nile. As has been demonstrated in other point-producing Levallois reduction systems [77], [78], the higher frequency of Nubian Type 1 cores may be linked to a greater emphasis on mobile hunting strategies, resulting in the frequent loss and needed replacement of Levallois points. The presence of numerous isolated Nubian Type 1 cores across the Nejd Plateau suggests that hunters carried them there to efficiently produce new points while far from sources of raw material and/or established camp sites.

## Discussion

The taxonomic identity of the Nubian Complex toolmakers is unknown, as no skeletal evidence has been discovered in association with any such assemblage. Although some archaic forms may have persisted in other parts of Africa at that time [79], the distribution of early anatomically modern human (AMH) remains suggest this species is the most likely candidate to have occupied northeast Africa during the Late Pleistocene. Cranial fragments of *Homo sapiens* found in the Omo river valley, Ethiopia (Fig. 1), represent the first appearance of AMH in East Africa ∼195 ka [80]. Remains from Herto [81], Singa [82], and Mumba [83] in East Africa date to between ∼160 and ∼100 ka. Skeletal remains from Jebel Irhoud in Morocco show that an early form of *Homo sapiens* had expanded into North Africa as early as ∼160 ka [84], and a modern human child discovered at Grotte des Contrebandiers in Morocco verifies the presence of AMH in North Africa by ∼110 ka [85]. At the site of Taramsa Hill 1 in the lower Nile Valley, an AMH child dated to ∼55 ka was found in association with a lithic industry (Taramsan) that is thought to have developed out of the late Nubian Complex [21], [86]. Despite the lack of direct evidence, given that AMH are the only species to have been found in North Africa from the late Middle Pleistocene onward, it is warranted to speculate that the Nubian Complex toolmakers were modern humans.

If MSA inhabitants of northeast Africa were AMHs, then the presence of a regionally-specific African MSA industry in Dhofar is relevant to the question of modern human expansion. The route and timing of *Homo sapiens* exit(s) from Africa is the subject of considerable debate [86]–[89]. Two pathways are commonly considered: the northern dispersal route postulates population movement from northeast Africa across the Sinai Peninsula into the Levant through the ‘Levantine Corridor.’ Alternatively (or concurrently), the southern dispersal route describes a demographic expansion through the ‘Arabian Corridor’, from the Horn of Africa across the southern Red Sea into Yemen.

Movement through the northern dispersal route is based on AMH remains discovered at Skhul and Qafzeh in Israel dating to early MIS 5 [90], [91]. Comparison of MSA/MP and LSA/UP lithic assemblages between northeast Africa and the Levant, however, does not reveal any evidence of cultural exchange. Marks [92] observes that the archaeological sequences from these two regions follow separate trajectories of development, suggesting there was no exchange of technologies. Vermeersch [12] arrives at a similar conclusion: “in the cultural material [of Egypt] no connections with the Levant are apparent.”

Genetic studies of human mtDNA favor the southern dispersal route as the primary conduit for early modern human expansion(s) out of Africa [93]–[97]. All non-Africans derive exclusively from basal mtDNA haplogroup L3 in Africa, which gave rise to descendant lineages M and N outside of Africa [98]. Haplogroups M and N are present in South and East Asia, Australia, and the Americas, but M lacks deep roots in western Eurasia [94]. This geographic patterning is most likely to have arisen if the first successful pioneers of the extant non-African population moved through Arabia and subsequently diversified in or east of the Peninsula.

To some degree, the discovery of late Nubian Complex assemblages in Dhofar upholds this model. The distribution of this technocomplex in the middle and lower Nile Valley, the Horn of Africa, Yemen, and now Dhofar provides a trail of diagnostic artifacts - stone breadcrumbs - spread across the southern dispersal route out of Africa. The close similarity between African and Arabian late Nubian Complex assemblages suggests that these sites are more or less contemporaneous; they were separated for an insufficient amount of time for independently derived technological traits to develop between regions. As the late Nubian Complex at Aybut Al Auwal is dated to MIS 5c, slightly earlier than the late Nubian Complex in Africa [11], we remain open to the possibility that the late Nubian Complex originated in Arabia, and subsequently spread back into northeast Africa. Given the coarse chronological resolution in both Africa and Arabia (Table 1), however, the question of directionality cannot be adequately addressed, suffice to say there is cultural exchange across the Red Sea during MIS 5c.

Coalescence ages for non-African mtDNA lineages range from 70 to 45 ka, depending on the use of different mutation rates, calibration methods, and statistical models [95], [99], placing these mtDNA studies at odds with the archaeological picture beginning to emerge from Arabia. We consider three possible explanations to reconcile the younger mtDNA and older archaeological evidence. First, groups moving out of Africa during MIS 5 may have carried older mtDNA types, such as L3′4′6′ [98]. Subsequent population bottlenecks from MIS 4 to MIS 2 are likely to have culled most of the founding populations in Arabia, which might be consistent with the rare presence of undifferentiated L3* lineages in Yemen [100]. Moreover, traces of the primarily East African haplogroup L4 have been reported in southern Arabia, with coalescence age estimates around 95 ka [98]. Unfortunately, little is known of this clade at present; too few L4 haplotypes have been observed to draw any conclusive phylogeographic inferences.

A second possibility is that the mtDNA coalescence age of L3 would appear younger than the time of initial expansion if pioneering groups moving into Arabia had been sex-biased toward a low number of females [101]. Finally, it may be the case that the Nubian Complex population did not expand past Dhofar and did not survive in Arabia over the course of the Late Pleistocene; hence, it is not represented in the extant genetic record.

The archaeological evidence does not yet permit us to evaluate what became of the late Nubian Complex in Arabia. Our study only documents the presence of this industry in Dhofar during MIS 5c; we do not yet know when Nubian Complex toolmakers arrived on the subcontinent or what became of them over the course of the Late Pleistocene. The eastern distribution of the Nubian Complex appears to terminate at the edge of Nejd plateau. Surveys throughout the rest of Oman and eastern Arabia have not produced any evidence of Nubian Complex technology. Assemblage C from the last interglacial site of Jebel Faya is classified as a generalized East African MSA technological complex (i.e., the concurrence of preferential centripetal Levallois with hard hammer blade and bifacial reduction) and is ascribed to AMH toolmakers. Its small assemblage size and limited workshop characteristics, however, preclude attribution to any specific, contemporaneous East African industry [71]. There are no characteristics, in terms of technology or typology, that overlap with the late Nubian Complex. Nor do the MP surface scatters from Sharjah, Ras Al Khaimah [69] and Abu Dhabi [22], also characterized by radial Levallois and bifacial reduction, share any affinities with the late Nubian Complex. The site of Jebel Qattar 1 in northern Saudi Arabia, which was excavated within an ancient lakeshore deposit dated to 75±5 ka, yielded centripetal preferential Levallois, radial, and bifacial technologies [74], while Nubian Levallois reduction is absent. As such, the Jebel Qattar 1 assemblage is much closer to MP assemblages found along the Gulf coast in eastern Arabia. Considering these broadly different technological packages found in the Arabian Peninsula during MIS 5, we surmise that at least two technologically (hence culturally) differentiated groups were present at this time: Nubian Levallois in southern Arabia and centripetal preferential Levallois with bifacial tools in northern/eastern Arabia. This observation may be relevant to discussions of admixture during the earliest phases of the human expansion [79], [102], [103].

The presence of seemingly Nubian-derived assemblages around the Wadi Aybut-Banut-Amut-Ghadun drainage systems, discovered during the DAP 2011 fieldwork campaign, hints at the survival of some aspects of the Nubian Complex technological tradition within Dhofar. These ‘Developed Nubian’ assemblages exhibit a suite of core reduction strategies including Nubian Levallois, ‘microlithic’ Nubian, and flat cores with bidirectional blades struck from faceted platforms. Such assemblages, however, must still be adequately defined and placed within a chronological framework.

Although southern Arabia experienced successive periods of extreme aridity after MIS 5, terrestrial archives document another increase in precipitation across the interior of Arabia during early MIS 3 [59], [104], enabling north-south demographic exchange between ∼60–50 ka. South Arabian populations may have spread to the north at this time, taking with them a Nubian-derived Levallois technology based on elongated point production struck from bidirectional Levallois cores, which is notably the hallmark of the Middle-Upper Palaeolithic transition in the Levant [105], [106]. Further survey in central Arabia is required to test whether the Nubian Complex extends north of Dhofar. Until then, the fate of the Nubian Complex in Arabia must remain in question.

## Supporting Information

Figure S1 **Example OSL decay and dose-response curves from AYB1-OSL1.** Decay curve (a) and dose-response curve (b) for a single aliquot of quartz (∼50 grains). The D_e_ of ∼70 Gy is obtained by interpolation of the sensitivity-corrected natural OSL signal, shown in red on the *y*-axis of the inset plot. The data in (a) and (b) were collected after preheating the natural and regenerative doses at 260°C for 10 s. Panel (c) shows the D_e_ values obtained from aliquots preheated at a range of temperatures (200–280°C for 10 s, with four replicates at each temperature), along with the extent of recuperation (i.e., the sensitivity-corrected OSL intensity at zero regenerative dose expressed as a percentage of the sensitivity-corrected natural OSL intensity); these data indicate that the measured D_e_ value is not sensitive to the chosen preheat temperature. The D_e_ values obtained from 42 separate aliquots of AYB1-OSL1 are displayed in (d); each aliquot was preheated at 260°C for 10 s. The filled circles and open triangles denote the values obtained using the ‘late light’ and ‘early background’ subtraction approaches, respectively, and the shaded band is centred on the weighted mean D_e_ value (∼58 Gy) used to calculate the OSL age of this sample. Plot (e) shows the D_e_ values obtained from 22 single aliquots of AYB1-OSL2: the symbols are the same as in (d) and the shaded band is centred on the weighted mean D_e_ value (∼61 Gy) used to estimate the sample age.
(TIF)

Table S1 **Equivalent dose (D_e_) values, environmental dose rates, and OSL ages of the sediment samples from Aybut Al Auwal.** Values are mean ± total (1σ) uncertainty, calculated as the quadratic sum of the random and systematic uncertainties. The D_e_ uncertainty includes a relative error of 2% to allow for possible bias in the calibration of the laboratory beta source.
(DOC)

Appendix S1 **Optically stimulated luminescence (OSL) dating.**
(DOC)
