# Supplementary figures and images for: The Nubian Complex of Dhofar, Oman: An African Middle Stone Age Industry in Southern Arabia
Source: PLoS One. 2011 Nov 30;6(11):e28239. doi: 10.1371/journal.pone.0028239 (PMC3227647; doi:10.1371/journal.pone.0028239)

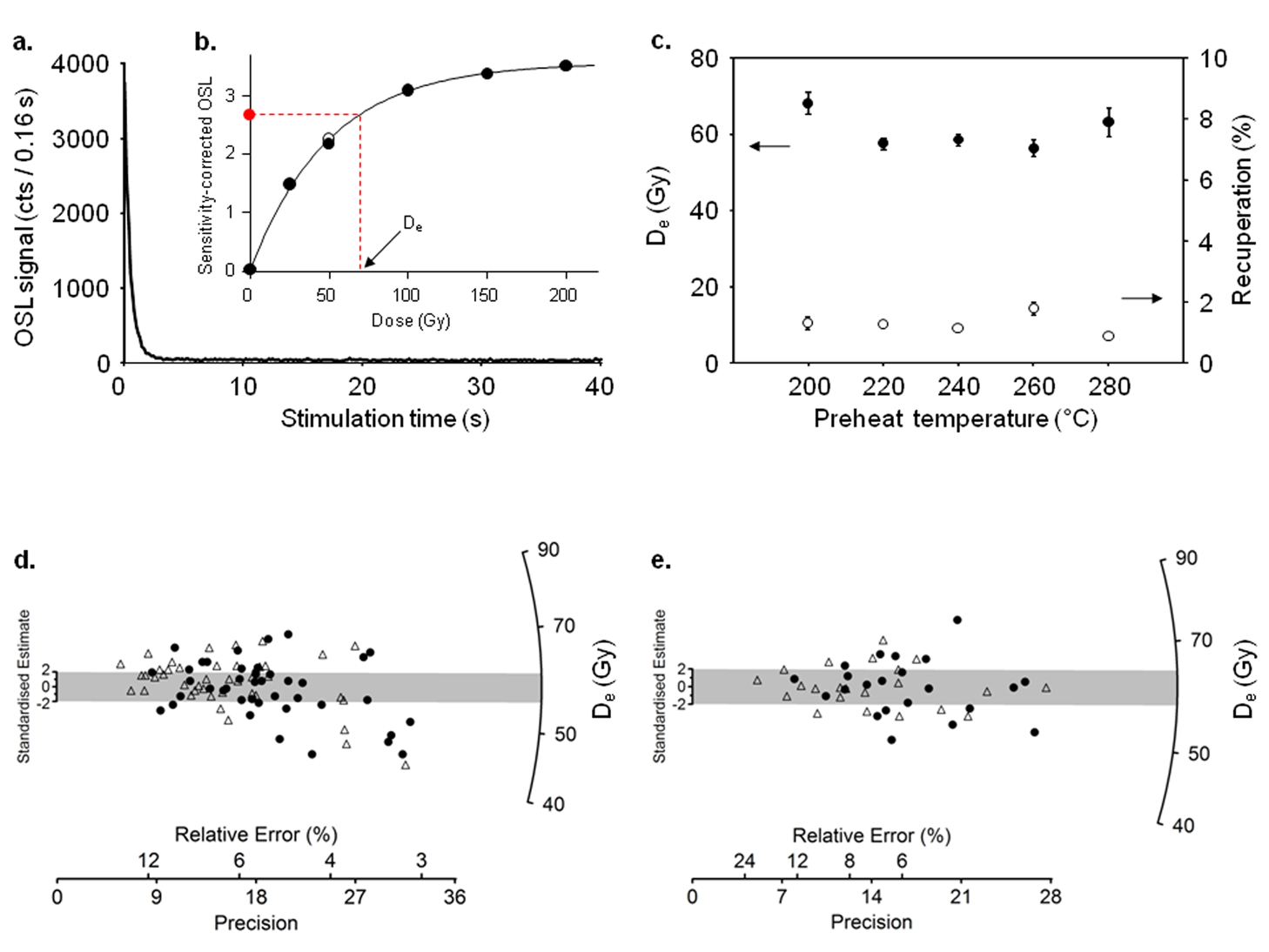

Supplement: Figure S1 — Example OSL decay and dose-response curves from AYB1-OSL1. Decay curve (a) and dose-response curve (b) for a single aliquot of quartz (∼50 grains). The De of ∼70 Gy is obtained by interpolation of the sensitivity-corrected natural OSL signal, shown in red on the y-axis of the inset plot. The data in (a) and (b) were collected after preheating the natural and regenerative doses at 260°C for 10 s. Panel (c) shows the De values obtained from aliquots preheated at a range of temperatures (200–280°C for 10 s, with four replicates at each temperature), along with the extent of recuperation (i.e., the sensitivity-corrected OSL intensity at zero regenerative dose expressed as a percentage of the sensitivity-corrected natural OSL intensity); these data indicate that the measured De value is not sensitive to the chosen preheat temperature. The De values obtained from 42 separate aliquots of AYB1-OSL1 are displayed in (d); each aliquot was preheated at 260°C for 10 s. The filled circles and open triangles denote the values obtained using the ‘late light’ and ‘early background’ subtraction approaches, respectively, and the shaded band is centred on the weighted mean De value (∼58 Gy) used to calculate the OSL age of this sample. Plot (e) shows the De values obtained from 22 single aliquots of AYB1-OSL2: the symbols are the same as in (d) and the shaded band is centred on the weighted mean De value (∼61 Gy) used to estimate the sample age. (TIF) [file pone.0028239.s001.tif]
