# Supplementary material for: The Nubian Complex of Dhofar, Oman: An African Middle Stone Age Industry in Southern Arabia
Source: PLoS One. 2011 Nov 30;6(11):e28239. doi: 10.1371/journal.pone.0028239 (PMC3227647; doi:10.1371/journal.pone.0028239)
Supplement: Table S1 — Equivalent dose (De) values, environmental dose rates, and OSL ages of the sediment samples from Aybut Al Auwal. Values are mean ± total (1σ) uncertainty, calculated as the quadratic sum of the random and systematic uncertainties. The De uncertainty includes a relative error of 2% to allow for possible bias in the calibration of the laboratory beta source. (DOC) [file pone.0028239.s002.doc]

Sample Depth Field water ————————————— Dose rates (Gy/ka) ————————————— D_e_ Age

code (m) content (%) Beta Gamma Internal Cosmic Total (Gy) (ka)

AYB1-OSL1 0.52 7.2 0.216 ± 0.011 0.124 ± 0.012 0.03 ± 0.01 0.177 ± 0.019 0.55 ± 0.04 58.0 ± 2.2 106 ± 9

AYB1-OSL2 0.74 5.1 0.229 ± 0.013 0.136 ± 0.014 0.03 ± 0.01 0.172 ± 0.018 0.57 ± 0.04 60.9 ± 2.7 107 ± 9
