# Supplementary material for: The Nubian Complex of Dhofar, Oman: An African Middle Stone Age Industry in Southern Arabia
Source: PLoS One. 2011 Nov 30;6(11):e28239. doi: 10.1371/journal.pone.0028239 (PMC3227647; doi:10.1371/journal.pone.0028239)
Supplement: Appendix S1 — Optically stimulated luminescence (OSL) dating. (DOC) [file pone.0028239.s003.doc]

**Appendix S1**

Optically stimulated luminescence (OSL) dating

OSL dating provides an estimate of the time elapsed since luminescent minerals, such as quartz, were last exposed to sunlight [1-4]. Buried grains are exposed to an ionizing radiation flux (the ‘dose rate’) from the surrounding sediments and from cosmic rays, resulting in the absorption of energy and storage of trapped charge. The corresponding ‘equivalent dose’ (D_e_) can be estimated using the OSL signal, and the burial age then obtained by dividing the D_e_ by the environmental dose rate. Table S1 lists the data used to determine the dose rates, D_e_ values and burial ages of the two sediment samples collected from Aybut Al Auwal (AYB1-OSL1 and AYB1-OSL2).

Large blocks of sediment (15 x 15 x 5 cm) were collected in daylight from depths of ~0.52 m (AYB1-OSL1) and ~0.74 m (AYB1-OSL2), and transported intact to Australia for OSL dating; subsequent excavations revealed a Nubian core buried close (~15 cm) to AYB1-OSL1. In the laboratory, we removed ~1 cm from all sides of both blocks under dim red illumination, to ensure that the external (sun-bleached) portions were not processed for dating. We then extracted quartz grains of 90–125 μm in diameter from the internal (light-safe) portion of each block using standard preparation procedures [2]. These included the successive application of hydrochloric acid (to dissolve carbonates), hydrogen peroxide (to oxidize any organic matter), dry sieving (to obtain different grain-size fractions) and density separation using sodium polytungstate solutions (to isolate the quartz from the heavy minerals and feldspars). The separated quartz grains were etched with 48% hydrofluoric acid for 40 min to remove the alpha-dosed rind of each quartz grain and destroy any remaining feldspars.

To check that the quartz grains had been sufficiently bleached at deposition, D_e_ values were obtained from small aliquots (each composed of ~50 grains) using the single-aliquot regenerative-dose (SAR) procedure, statistical models and experimental apparatus described elsewhere [5-7]. The bleaching and post-depositional history of a sample can be assessed using small aliquots by examining the distribution pattern of D_e_ values and quantifying the amount of scatter [3-8].

Forty-eight aliquots of each sample were optically stimulated by blue (470 nm) light for 40 s at 125°C, after preheating the natural and regenerative doses at 260°C for 10 s (or at 200°C for 5 s for the test doses used to correct for any sensitivity changes) and exposing the grains to 40 s of infrared (IR) radiation at 50°C [5]. The ultraviolet OSL emissions were detected using an EMI 9635Q photomultiplier tube fitted with Hoya U-340 filters, and laboratory doses were given using a calibrated ^90^Sr/^90^Y beta source. The IR stimulation was used to minimize any shortfall in D_e_ associated with the malign effects of fading that afflicts feldspars. The latter may occur as impurities within etched quartz grains, but the weak IR stimulated signals emitted during the SAR measurement sequence suggests that feldspar contamination is negligible in these samples.

The D_e_ of each aliquot was calculated from the first 0.8 s of the OSL signal, after subtracting a (‘late light’) background count rate from the final 8 s of stimulation. Each aliquot exhibited a rapid rate of initial OSL decay (consistent with the signals arising from the most light-sensitive (‘fast’) component of quartz OSL) and the dose-response data were fitted using a saturating exponential plus linear function [2]. A representative OSL decay curve and dose-response curve are shown in Figures S1a and S1b, respectively. The D_e_ was obtained by projecting the sensitivity-corrected natural OSL signal on to the fitted dose-response curve, and the uncertainty on this estimate (due to photon counting statistics, curve-fitting uncertainties, and an allowance of 1% per OSL measurement for instrument irreproducibility) was determined by Monte Carlo simulation, using the procedures described in Duller [9] and implemented in *Analyst* version 3.24.

We also calculated the D_e_ values using an ‘early background’ subtraction procedure (using the counts in the 0.8–1.6 s interval), which has been proposed as a means of minimizing any influence from the less light-sensitive (‘medium’ and ‘slow’) components of quartz OSL [10], [11]. For both samples, we found no significant difference in the range of D_e_ values obtained using these alternative methods of background subtraction (Figures S1d and S1e), or in their weighted mean D_e_ values. For AYB1-OSL1, late light subtraction resulted in a weighted mean D_e_ of 58.0 ± 1.8 Gy, compared to the value of 62.2 ± 2.0 Gy obtained using early background subtraction; the corresponding weighted means for AYB1-OSL2 are 60.9 ± 2.4 Gy and 61.3 ± 2.2 Gy, respectively. These results confirm that the initial OSL signal is dominated by the ‘fast’ component.

We conducted standard quality assurance tests of SAR procedure performance [4], [5], [7], including checks for thermal transfer (negligible recuperation at zero applied dose) and adequate sensitivity correction (‘recycling’ ratios of 0.9–1.1 for sensitivity-corrected OSL signals from duplicate regenerative doses given at the start and end of the measurement sequence). These tests revealed no significant problems for 42 of the 48 aliquots of AYB1-OSL1; the remaining six aliquots of this sample had dose-response curves that saturated at low doses, so finite D_e_ values could not be obtained. For sample AYB1-OSL2, a larger number of aliquots saturated at low doses or failed to satisfy other quality assurance criteria, so reliable estimates of D_e_ could be obtained from only 22 of the 48 aliquots.

Prior to making these D_e_ measurements, we performed a preheat plateau test [2] on 20 natural aliquots (each consisting of ~500 grains) of AYB1-OSL1. The natural and regenerative doses were preheated at 200, 220, 240, 260 or 280°C for 10 s, and no dependency in D_e_ or the extent of recuperation (which amounted to only 1–2% of the natural OSL intensity) was observed across this temperature range (Figure S1c). We also conducted a dose recovery test [5] on 12 natural aliquots of AYB1-OSL1: these were bleached in sunlight for 2 days, given a laboratory beta dose of 52 Gy as a surrogate for the natural dose, and then measured using a preheat of 260°C for 10 s. The mean ratio of measured dose to given dose of 0.99 ± 0.03 demonstrates that a known dose similar in size to the sample D_e_ can be accurately recovered under controlled laboratory conditions using the chosen experimental parameters.

Figure S1d is a radial plot [5] of the D_e_ values obtained from 42 aliquots of AYB1-OSL1, and Figure S1e shows the 22 independent estimates of D_e_ obtained for AYB1-OSL2. The D_e_ for a single aliquot is read by drawing a line from the origin of the standardized estimate axis, through the point of interest, until it intersects the D_e_ axis. The uncertainty on this D_e_ is obtained by drawing a vertical line from the data point to intersect the relative error axis. Any shaded band of ±2 units projecting from the standardized estimate axis should capture 95% of the points if the measurement uncertainties are sufficient to account for the observed scatter in D_e_. Clearly, the D_e_ values are spread more widely than can be explained by their measurement uncertainties alone, which is the norm for single grains and multi-grain aliquots of natural quartz [3], [4], [12].

The relative spread in D_e_ values between aliquots, beyond that associated with the individual measurement uncertainties, is commonly referred to as ‘overdispersion’ [5], [13], [14]. The overdispersion estimates of 19.4 ± 2.3% (AYB1-OSL1) and 16.9 ± 2.9% (AYB1-OSL2) fall within the range of values reported previously for single aliquots of quartz that are known or thought to have been fully bleached at burial and not disturbed by post-depositional mixing [12], [13]. The distribution pattern of D_e_ values also shows no evidence for incomplete bleaching of the grains before deposition: the latter commonly results in a significantly positively-skewed distribution, with a concentration of values at low doses and a ‘tail’ of higher D_e_ values [4], [6], [8], [15]. Neither of the Aybut Al Auwal samples exhibits this distribution pattern, and application of a weighted skewness test [12], [16] indicates that the distribution of log D_e_ values is not significantly skewed (AYB1-OSL1: *P* = 0.114; AYB1-OSL2: *P* = 0.589).

As shown by a factorial experiment [13] and by other controlled trials [14], [17], [18], there are many possible causes of D_e_ overdispersion that cannot be completely accounted for by experimental measurements in the laboratory, but which are unrelated to field complications such as incomplete bleaching, post-depositional mixing or variations in the beta dose rate to individual grains. For example, non-identical field and laboratory conditions include differences in the intensity and wavelength of the bleaching spectra, the type of ionizing radiation and the rate at which dose is delivered to the grains, and the length of time available for any charge redistribution due to defect migration [13]. Also, the heating and bleaching procedures employed in the SAR measurement sequence will likely not empty the OSL source traps in all grains uniformly, and thermal transfer effects are apt to vary between grains. The results of the dose recovery test provide direct evidence of such inherent ‘natural’ variability: the measured doses were overdispersed by ~8%, despite having eliminated any field-related problems by exposing the grains to prolonged sunlight before administering an identical dose in the laboratory. Given the similarities between the D_e_ distribution patterns and overdispersion values of the Aybut Al Auwal samples and those of other well-bleached and undisturbed samples of natural quartz, we estimated their burial doses from the weighted mean D_e_. We calculated the latter using the central age model of Galbraith et al. [5]; the associated standard errors take into account all measurement uncertainties and the additional spread due to D_e_ overdispersion.

The environmental dose rates were calculated as the sum of the beta and gamma dose rates due to the radioactive decay of ^238^U, ^235^U, ^232^Th (and their daughter products) and ^40^K, plus the contribution from cosmic rays. We also included an effective internal alpha dose rate of 0.03 ± 0.01 Gy/ka, which captures the range of values (0.01–0.05 Gy/ka) measured previously for sedimentary quartz grains from Australia and Africa [18-20]. The beta dose rates were determined from a dried and powdered portion of each sample using a Risø GM-25-5 low-level beta counter, making allowance for beta dose attenuation due to grain size [21] and hydrofluoric acid etching [22]. The gamma dose rates were measured in the field to take account of any spatial heterogeneity in the gamma radiation field surrounding each sample. A hand-auger was used to drill 30 cm into the deposit at both sample locations and the NaI(Tl) detector was inserted into these holes. The spectrometer was calibrated using the concrete blocks at Oxford [23] and the gamma dose rates were determined using the ‘threshold’ technique [24]. By measuring the beta and gamma dose rates using these techniques, we have implicitly assumed that the present-day state of (dis)equilibrium in the uranium and thorium decay chains has prevailed throughout the period of sample burial. Modelling has shown, however, that this assumption is unlikely to lead to errors in the dose rate of more than 2–3%, even for the most common time-dependent disequilibria in the ^238^U series, when such techniques are employed [25], [26].

The beta and gamma dose rates were calculated for a sample water content of 8 ± 4%, which accommodates the measured water contents of both Aybut Al Auwal samples (Table S1) and the likely variations averaged over the entire period of sample burial. The latter encompasses the last glacial period (which was more arid than present), so it would be unreasonable to assign a much higher long-term value; a much lower value for the water content is also not realistic, as the sample was collected during the dry season. The calculated ages are not particularly sensitive to the chosen value, however, increasing only by ~1% for each 1% increase in water content. The cosmic-ray dose rates were estimated from published equations [27], taking into account the latitude, longitude and altitude of Aybut Al Auwal and long-term changes in the geomagnetic field, as well as the water contents of the samples [28] and their depths of burial, both averaged over the time since deposition. AYB1-OSL1 and AYB1-OSL2 were collected from depths of ~0.52 m and ~0.74 m, respectively, and have probably never been buried deeper than ~3 m, given the geomorphic context of the site and the elevation of the surrounding higher terraces. As a compromise, we calculated the OSL ages of these samples assuming time-averaged burial depths of 1 m (AYB1-OSL1) and 1.2 m (AYB1-OSL2), and assigned each a relative uncertainty of ± 25% to cover plausible alternative scenarios. The total uncertainties on the resulting ages (AYB1-OSL1: 106 ± 9 ka; AYB1-OSL2: 107 ± 9 ka) comfortably includes the estimates of ~103 and ~105 ka obtained for these samples under the assumption that they have been buried at their present depths since deposition.

The OSL ages for AYB1-OSL1 and AYB1-OSL2 are statistically indistinguishable and give a weighted mean age of 106.6 ± 6.4 ka for the dated grains at Aybut Al Auwal; this age is consistent with sediment deposition during Marine Isotope Stage 5c.

**Appendix S1 References**

1. Huntley DJ, Godfrey-Smith DI, Thewalt MLW (1985) Optical dating of sediments. Nature 313: 105–107.
2. Aitken MJ (1998) An Introduction to Optical Dating: the dating of Quaternary sediments by the use of photon-stimulated luminescence. Oxford: Oxford University Press.
3. Lian OB, Roberts RG (2006) Dating the Quaternary: progress in luminescence dating of sediments. Quatern Science Rev 25: 2449–2468.
4. Jacobs Z, Roberts RG (2007) Advances in optically stimulated luminescence dating of individual grains of quartz from archeological deposits. Evol Anthropol 16: 210–223.
5. Galbraith RF, Roberts RG, Laslett GM, Yoshida H, Olley JM (1999) Optical dating of single and multiple grains of quartz from Jinmium rock shelter, northern Australia: Part I, experimental design and statistical models. Archaeometry 41: 339–364.
6. Olley JM, Pietsch T, Roberts RG (2004) Optical dating of Holocene sediments from a variety of geomorphic settings using single grains of quartz. Geomorph 60: 337–358.
7. Jacobs Z, Roberts RG, Galbraith RF, Deacon HJ, Grün R, et al. (2008) Ages for the Middle Stone Age of southern Africa: implications for human behavior and dispersal. Science 322: 733–735.
8. Olley JM, Caitcheon GG, Roberts RG (1999) The origin of dose distributions in fluvial sediments, and the prospect of dating single grains from fluvial deposits using optically stimulated luminescence. Radiation Measurements 30: 207–217.
9. Duller GAT (2007) Assessing the error on equivalent dose estimates derived from single aliquot regenerative dose measurements. Ancient TL 25: 15–24.
10. Cunningham AC, Wallinga J (2010) Selection of integration time intervals for quartz OSL decay curves. Quatern Geochronology 5: 657–666.
11. Pawley SM, Toms P, Armitage SJ, Rose J (2010) Quartz luminescence dating of Anglian Stage (MIS 12) fluvial sediments: comparison of SAR age estimates to the terrace chronology of the Middle Thames valley, UK. Quatern Geochronology 5: 569–582.
12. Arnold LJ, Roberts RG (2009) Stochastic modelling of multi-grain equivalent dose (De) distributions: implications for OSL dating of sediment mixtures. Quatern Geochronology 4: 204–230.
13. Galbraith RF, Roberts RG, Yoshida H (2005) Error variation in OSL palaeodose estimates from single aliquots of quartz: a factorial experiment. Radiation Measurements 39: 289–307.
14. Roberts RG, Galbraith RF, Yoshida H, Laslett GM, Olley JM (2000) Distinguishing dose populations in sediment mixtures: a test of single-grain optical dating procedures using mixtures of laboratory-dosed quartz. Radiation Measurements 32: 459–465.
15. Arnold LJ, Roberts RG, Galbraith RF, DeLong SB (2009) A revised burial dose estimation procedure for optical dating of young and modern-age sediments. Quatern Geochronology 4: 306–325.
16. Bailey RM, Arnold LJ (2006) Statistical modelling of single grain quartz De distributions and an assessment of procedures for estimating burial dose. Quatern Science Rev 25: 2475–2502.
17. Thomsen KJ, Murray AS, Bøtter-Jensen L (2005) Sources of variability in OSL dose measurements using single grains of quartz. Radiation Measurements 39: 47–61.
18. Jacobs Z, Duller GAT, Wintle AG, Henshilwood CS (2006) Extending the chronology of deposits at Blombos Cave, South Africa, back to 140 ka using optical dating of single and multiple grains of quartz. J Hum Evol 51: 255–273.
19. Feathers JK, Migliorini E (2001) Luminescence dating at Katanda – a reassessment. Quatern Science Rev 20: 961–966.
20. Bowler JM, Johnston H, Olley JM, Prescott JR, Roberts RG, et al. (2003) New ages for human occupation and climatic change at Lake Mungo, Australia. Nature 421: 837–840.
21. Mejdahl V (1979) Thermoluminescence dating: beta-dose attenuation in quartz grains. Archaeometry 21: 61–72.
22. Bell WT, Zimmerman TW (1978) The effect of HF acid etching on the morphology of quartz inclusions for thermoluminescence dating. Archaeometry 20: 63–65.
23. Rhodes EJ, Schwenninger J-L (2007) Dose rates and radioisotope concentrations in the concrete calibration blocks at Oxford. Ancient TL 25: 5–9.
24. Mercier N, Falguères C (2007) Field gamma dose-rate measurement with a NaI(TI) detector: re-evaluation of the “threshold” technique. Ancient TL 25: 1–4.
25. Olley JM, Murray A, Roberts RG (1996) The effects of disequilibria in the uranium and thorium decay chains on burial dose rates in fluvial sediments. Quatern Science Rev 15: 751–760.
26. Olley JM, Roberts RG, Murray AS (1997) Disequilibria in the uranium decay series in sedimentary deposits at Allen’s Cave, Nullarbor Plain, Australia: implications for dose rate determinations. Radiation Measurements 27: 433–443.
27. Prescott JR, Hutton JT (1994) Cosmic ray contributions to dose rates for luminescence and ESR dating: large depths and long-term time variations. Radiation Measurements 23: 497–500.
28. Readhead ML (1987) Thermoluminescence dose rate data and dating equations for the case of disequilibrium in the decay series. Nuclear Tracks and Radiation Measurements 13: 197–207.
